# Genomic and Transcriptional Alterations in Lung Adenocarcinoma in Relation to *EGFR* and *KRAS* Mutation Status

**DOI:** 10.1371/journal.pone.0078614

**Published:** 2013-10-24

**Authors:** Maria Planck, Karolina Edlund, Johan Botling, Patrick Micke, Sofi Isaksson, Johan Staaf

**Affiliations:** 1 Department of Oncology, Clinical Sciences, Lund University and Skåne University Hospital, Medicon Village, Lund, Sweden; 2 Department of Immunology, Genetics and Pathology, Uppsala University, Uppsala, Sweden; 3 CREATE Health Strategic Center for Translational Cancer Research, Lund University, Medicon Village, Lund, Sweden; Duke-National University of Singapore Graduate Medical School, Singapore

## Abstract

**Introduction:**

In lung adenocarcinoma, the mutational spectrum is dominated by *EGFR* and *KRAS* mutations. Improved knowledge about genomic and transcriptional alterations in and between mutation-defined subgroups may identify genes involved in disease development or progression.

**Methods:**

Genomic profiles from 457 adenocarcinomas, including 113 *EGFR*-mutated, 134 *KRAS*-mutated and 210 *EGFR* and *KRAS*-wild type tumors (EGFRwt/KRASwt), and gene expression profiles from 914 adenocarcinomas, including 309 *EGFR*-mutated, 192 *KRAS*-mutated, and 413 EGFRwt/KRASwt tumors, were assembled from different repositories. Genomic and transcriptional differences between the three mutational groups were analyzed by both supervised and unsupervised methods.

**Results:**

*EGFR*-mutated adenocarcinomas displayed a larger number of copy number alterations and recurrent amplifications, a higher fraction of total loss-of-heterozygosity, higher genomic complexity, and a more distinct expression pattern than *EGFR*-wild type adenocarcinomas. Several of these differences were also consistent when the three mutational groups were stratified by stage, gender and smoking status. Specific copy number alterations were associated with mutation status, predominantly including regions of gain with the highest frequency in *EGFR*-mutated tumors. Differential regions included both large and small regions of gain on 1p, 5q34-q35.3, 7p, 7q11.21, 12p12.1, 16p, and 21q, and losses on 6q16.3-q21, 8p, and 9p, with 20-40% frequency differences between the mutational groups. Supervised gene expression analyses identified 96 consistently differentially expressed genes between the mutational groups, and together with unsupervised analyses these analyses highlighted the difficulty in broadly resolving the three mutational groups into distinct transcriptional entities.

**Conclusions:**

We provide a comprehensive overview of the genomic and transcriptional landscape in lung adenocarcinoma stratified by *EGFR* and *KRAS* mutations. Our analyses suggest that the overall genomic and transcriptional landscape of lung adenocarcinoma is affected, but only to a minor extent, by *EGFR* and *KRAS* mutation status.

## Introduction

Lung cancer is a heterogeneous malignancy with poor survival due to diagnosis at an often advanced stage [1]. Lung cancer is broadly divided into small cell lung cancer (~15% of all lung cancers) and non-small cell lung cancer with adenocarcinoma as the most frequent histological type [2]. In adenocarcinoma, the mutational spectrum is dominated by *EGFR* and *KRAS* mutations, where the former is an established predictor of response to EGFR inhibitors [3,4]. *EGFR* and *KRAS* mutations are nearly always mutually exclusive and associated with differences in patient gender and smoking history [5]. Together, this suggests that these genetic alterations may be drivers of pathogenesis for specific adenocarcinoma subgroups [5] (and references therein). In *EGFR* and *KRAS*-wild type adenocarcinomas (EGFRwt/KRASwt), different potential drivers of pathogenesis exist, including *ALK*, *RET*, and *ROS1* gene fusions, with *ALK* rearrangements being therapeutically relevant [5-7]. Several studies have reported genomic or transcriptional alterations between *EGFR*-mutated and/or *KRAS*-mutated tumors and corresponding wild-type adenocarcinomas [8-18]. However, the majority of previous studies are based on relatively small patient cohorts and do not always stratify tumors into all three mutational groups, which may explain conflicting results. *EGFR*-mutated adenocarcinomas have repeatedly been associated with the bronchioid gene expression subtype originally defined by Hayes et al. [19]. Bronchioid classified tumors are generally of lower grade, have a higher expression of excretion, asthma and surfactant genes, occur predominantly in women and never-smokers, and have better overall survival compared with the other two expression subtypes, magnoid and squamoid [19,20]. The magnoid and squamoid subtypes harbor more *KRAS* mutations, seem to be more closely related in gene expression, occur more often in men and smokers, and have poorer overall survival [19,20].

**Figure 1:**
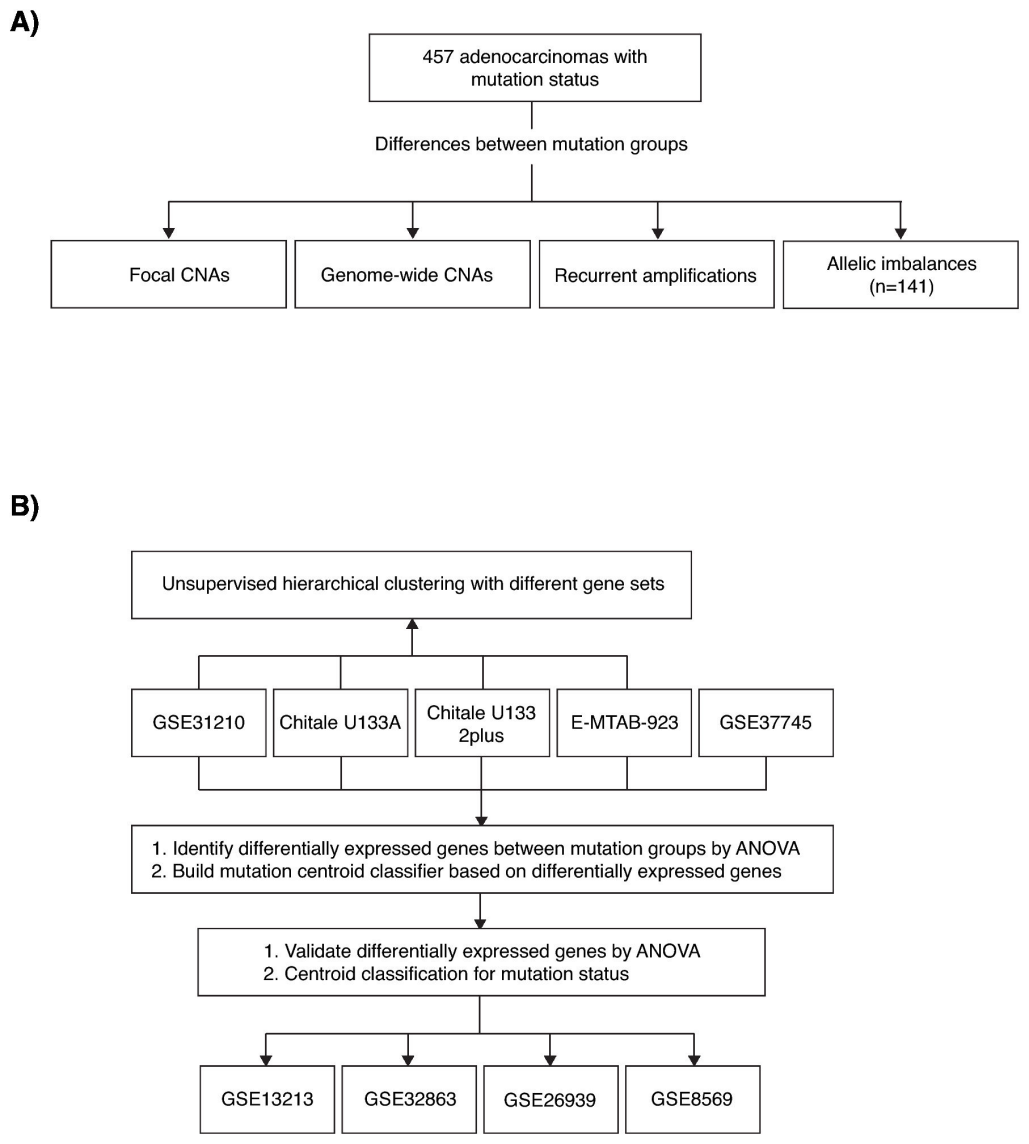
Schematic diagram of genomic and transcriptional analyses. (**A**) Genomic analyses. (**B**) Transcriptional analyses. Individual cohorts are portrayed.

To resolve conflicting reports and provide a comprehensive survey of copy number alterations, allelic imbalances and transcriptional alterations in lung adenocarcinomas stratified by *EGFR* and *KRAS* mutation status, we analyzed 457 genomic and 914 gene expression profiles for differences between the three mutational groups (Figure 1). We show that a few consistent genomic differences exist between the mutational groups, however with moderate frequencies. Transcriptional analyses identified only a small set of differentially expressed genes across multiple cohorts, and highlighted the difficulty to resolve the three mutational groups as distinct transcriptional entities. Together, our results suggest that the genomic and transcriptional landscape of lung adenocarcinoma is only to a minor extent determined by the mutational status of *EGFR* and *KRAS*.

## Materials and Methods

### Tumor material

Genomic profiles from 1272 adenocarcinoma tumors and cell lines were obtained from a previous study (n=1210) [21], with addition of adenocarcinomas from Wilkerson et al. [20] (n=62, GSE36363). All genomic profiles were analyzed in an unmatched fashion and sample uniqueness was assured as described [21]. All included tumors represented primary disease based on information from original studies. *EGFR* and *KRAS* mutational status was available for 457 adenocarcinoma, including 113 *EGFR*-mutated, 134 *KRAS*-mutated, and 210 EGFRwt/KRASwt tumors. 

Gene expression profiles from 914 adenocarcinoma tumors, including 309 *EGFR*-mutated, 192 *KRAS*-mutated and 413 EGFRwt/KRASwt cases, were collected from eight studies analyzed by different microarray platforms [8,10,20,22-26]. Samples from Chitale et al. [10] were further divided into two cohorts according to their different Affymetrix platforms (U133A and U133 2plus).

Explicit information on patient ethnicity or specific mutation type was not available for the majority of the included studies, and these parameters were therefore omitted from the analyses. However, the included studies were performed in both western and Asian countries. Patient and tumor characteristics are summarized in Tables 1 and 2, with additional description in File S1.

**Table 1:** Characteristics of individual aCGH and SNP genomic adenocarcinoma cohorts stratified by microarray platform.

Genomic cohort	No. of AC^A^	Microarray platform	Included in CN / GAP analysis^B^	No. of tumors / cell lines	No. of *EGFR*-mutated / *KRAS*-mutated / EGFRwt/KRASwt^C^	No. of stage I/II/III/IV tumors^C^	Gender female / male^C^	Smoking status NS/S^C,D^
Zhao [49]	36	Affymetrix 100K	Yes/No	36/0	-	-	2/1	-
GSE18252 [50]	4	Affymetrix 100K	Yes/Yes	4/0	0/4/0	-	-	-
Weir [51]	112	Affymetrix 250K Sty	Yes/No	112/0	-	-	-	-
Weir [51]	196	Affymetrix 250K Sty	Yes/No	196/0	15/49/51	41/14/15/3	110/83	17/116
GSE19399 [52]	19	Affymetrix 250K Sty	Yes/Yes	19/0	1/1/8	10/1/4/0	10/9	2/16
GSE17247 [53]	49	Affymetrix 250K Sty	Yes/No	0/49	-	-	-	-
GSE28572 [27]	44	Affymetrix 250K Nsp	Yes/Yes	44/0	9/18/17	21/10/8/2	27/17	4/37
GSE34140 [54]	141	Affymetrix 250K Nsp	Yes/No	141/0	-	79/26/9/7	-	10/107
GSK [55]	10	Affymetrix 250K Nsp	Yes/No	0/10	-	-	-	-
GSE19804 [56]	12	Affymetrix 6.0	Yes/No	12/0	-	6/3/2/1	12/0	12/0
GSE25016 [34]	58	Affymetrix 6.0	Yes/No	58/0	-	-	-	-
GSE33848 [9]	146	Affymetrix 6.0	Yes/No	146/0	-	95/0/0/0	-	-
TCGA-AC [57]	135	Affymetrix 6.0	Yes/No	135/0	-	73/23/27/9	78/57	19/109
GSE36363 [20]	62	Affymetrix 6.0 & 250K Nsp	Yes/Yes	62/0	5/14/24	39/8/11/0	37/25	5/56
Chitale [10]	184	Agilent 44K	Yes/No	184/0	42/45/97	121/26/32/5	107/77	39/145
GSE20393 [58]	2	Agilent 244K	Yes/No	2/0	-	-	-	-
E-TABM-926 [59]	17	Agilent 244K	Yes/No	17/0	13/1/3	8/1/8/0	14/3	17/0
E-TABM-1169 [59]	40	Illumina 370K	Yes/Yes	40/0	28/2/10	22/5/13/0	36/4	40/0
GSE31586	5	ROMA 85K	Yes/No	0/5	-	-	-	-
TOTAL	1272	-	1272/733	1208/64	113/134/210	515/117/129/27	433/276	165/586

A: Number of used adenocarcinoma cases per cohort.B: Included in overall GISTIC analysis (CN) and/or GAP-analysis for EGFR/KRAS mutation groups.C: For tumors only.

D: NS = never-smoker, S = smoker

**Table 2:** Clinical characteristics of patients with available mutation status in gene expression cohorts.

	**Discovery cohorts**					**Validation cohorts**			
	**GSE31210** [26]	**Chitale U133A** [10]	**Chitale U133 2plus** [10]	E-MTAB-923 [24]*	**GSE37745** [25]	**GSE13213** [22]	**GSE32863** [23]	GSE26939 [20]*	**GSE8569** [8]
**Total number of patients**	226	91	102	99	106	117	58	85	30
**Gender**									
Male	105	41	42	15	46	60	13	37	20
Female	121	50	60	84	60	57	45	48	10
**Mutation status**									
*EGFR*-mutated	127	15	24	49	18	45	17	11	3
*KRAS*-mutated	20	11	36	17	43	15	22	20	8
EGFRwt/KRASwt	79	65	42	33	45	57	19	54	19
**Stage**									
I	168	53	70	57	70	79	-	48	-
II	58	20	10	10	19	13	-	18	-
III	0	18	17	32	13	25	-	11	-
IV	0	0	5	0	4	0	-	3	-
**Usage**									
Differential gene expression	x	x	x	x	x	x	x	x	x
Unsupervised analysis	x	x	x	x					
**Platform**	Affymetrix U133 2plus	Affymetrix U133A	Affymetrix U133 2plus	Affymetrix U133 2plus	Affymetrix U133 2plus	Agilent 44K	Illumina WG6 V3	Agilent 44K	Custom cDNA

* These cohorts contain additional samples with unknown *EGFR* and *KRAS* mutation status.

### EGFR and KRAS mutation analysis

*EGFR* and *KRAS* mutation status was determined as described in either File S1 (for GSE37745 [25] and GSE28572 [27]) or in each of the original articles.

### Genomic analyses

Normalized copy number and B allele frequency estimates for Affymetrix microarrays and Illumina SNP beadchips, and normalized copy number estimates for Agilent 44K, Agilent 244K and ROMA 85K cohorts were generated and/or assembled as described in Staaf et al. [21] and File S1. Probe annotations for all array platforms were updated to the hg18/NCBI36 genome build. Genomic profiles were partitioned, centralized, and merged to a common probe set as described ([21] and File S1). 

A modified version of Genomic Identification of Significant Targets in Cancer (GISTIC) [28], referred to as mGISTIC herein, was used for identification of focal copy number alterations and recurrent amplifications from the 1272-sample cohort (see [21] and File S1). Robustness of identified regions was assessed by permutation analysis (Figure S1 and File S1). A genome-wide screen of differential copy number gain and loss between the three *EGFR* and *KRAS* defined mutation groups was performed by division of genomic profiles into 12,698 sequential segments of ~200 Kbp size, excluding reported regions of copy number variation. Each segment was subsequently tested for differences in frequency of copy number gain or loss individually. Fisher’s exact test or the Chi-square test was used to identify genomic regions and recurrent amplifications with different frequency between mutation groups. 

For tumors analyzed by SNP microarrays (n=141), B allele frequency estimates were partitioned [29], integrated with copy number data, and subjected to Genome Alteration Print (GAP) [30] analysis for estimation of allele-specific copy numbers and in silico tumor ploidy (referred to as GAP-ploidy herein) as described [21]. Loss-of-heterozygosity (LOH), copy-neutral LOH, and copy-neutral allelic imbalance were estimated from GAP results as described [21]. The fractions of the genome altered by copy number alterations, LOH, copy-neutral LOH, and copy-neutral allelic imbalance were calculated as described [21]. Data processing steps are further described in File S1 and [21].

### Gene expression analyses

Affymetrix cohorts were individually normalized using GC Robust Multi-array Averaging (GCRMA) [31]. For non-Affymetrix cohorts, normalized expression data were obtained from Gene Expression Omnibus [32]. In total, nine cohorts were analyzed individually for transcriptional differences between mutation groups as either discovery (n=5) or validation cohorts (n=4) (Table 2). Differentially expressed genes between *EGFR*-mutated, *KRAS*-mutated and EGFRwt/KRASwt tumors were identified by ANOVA with false discovery rate adjustment using a 5% threshold for statistical significance. Hierarchical clustering was performed using Pearson correlation and complete linkage. Data processing steps are further described in File S1.

## Results

### Copy number alterations in lung adenocarcinoma

**Figure 2:**
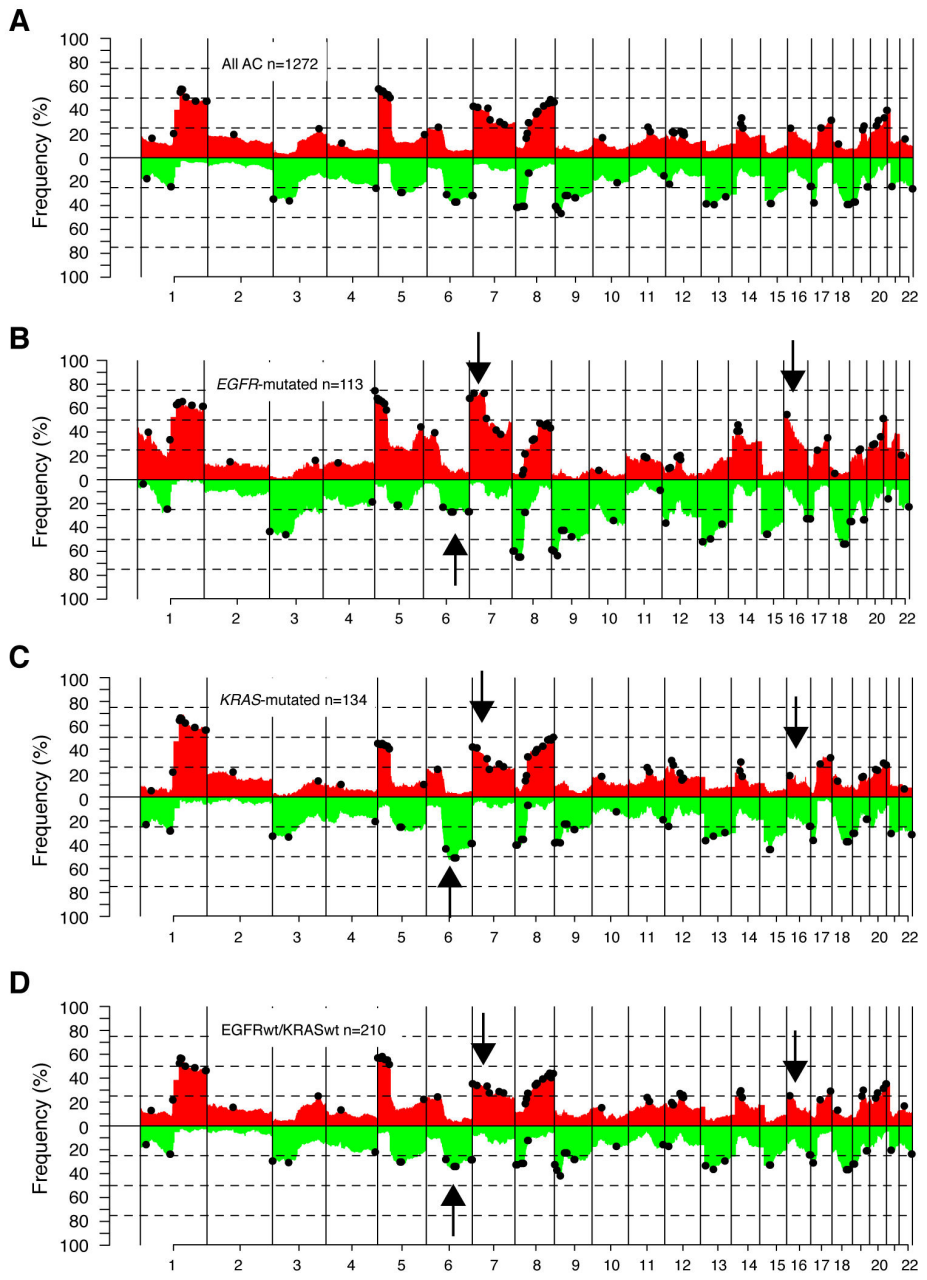
Copy number alterations in lung adenocarcinoma. Frequency of copy number gain (red) and loss (green) for adenocarcinoma stratified by *EGFR* and *KRAS* mutational status using log_2_ratio ± 0.12 as threshold for identification of copy number gain and loss. Probes matched to known copy number variations are excluded. Black regions indicate genomic position of significant mGISTIC regions, which were identified from analysis of the entire 1272-sample cohort across chromosomes. Arrows indicate genomic regions with apparently different copy number alteration frequency between EGFR/KRAS mutation groups (6q, 7p, and 16p). (**A**) All 1272 adenocarcinomas. (**B**) 113 *EGFR*-mutated adenocarcinoma tumors. (**C**) 134 *KRAS*-mutated adenocarcinoma tumors. (**D**) 210 EGFRwt/KRASwt adenocarcinoma tumors.

To identify copy number alterations (CNAs) of general importance in lung adenocarcinoma, which may serve as basis for supervised comparisons between *EGFR*-mutated, *KRAS*-mutated and EGFRwt/KRASwt tumors, we analyzed 1272 tumors and cell lines profiled by SNP or aCGH microarrays (Figure 2A, Table 1). To pinpoint recurrent CNAs in lung adenocarcinoma, we performed an mGISTIC analysis of the entire 1272-sample set identifying 59 gains and 31 losses distributed across all autosomes (Figure 2A, Table S1). Several of the identified mGISTIC regions harbored known or putative adenocarcinoma driver candidates, such as *EGFR*, *MDM2*, *KRAS*, *MYC*, *TERT*, *MET*, *CCND1*, *NKX2-1/TITF1*, *CDK4*, *ERBB2*, *ID1*, *RB1*, *CDKN2A* and *PTEN*.

### Copy number alterations in EGFR/KRAS mutation groups

**Figure 3:**
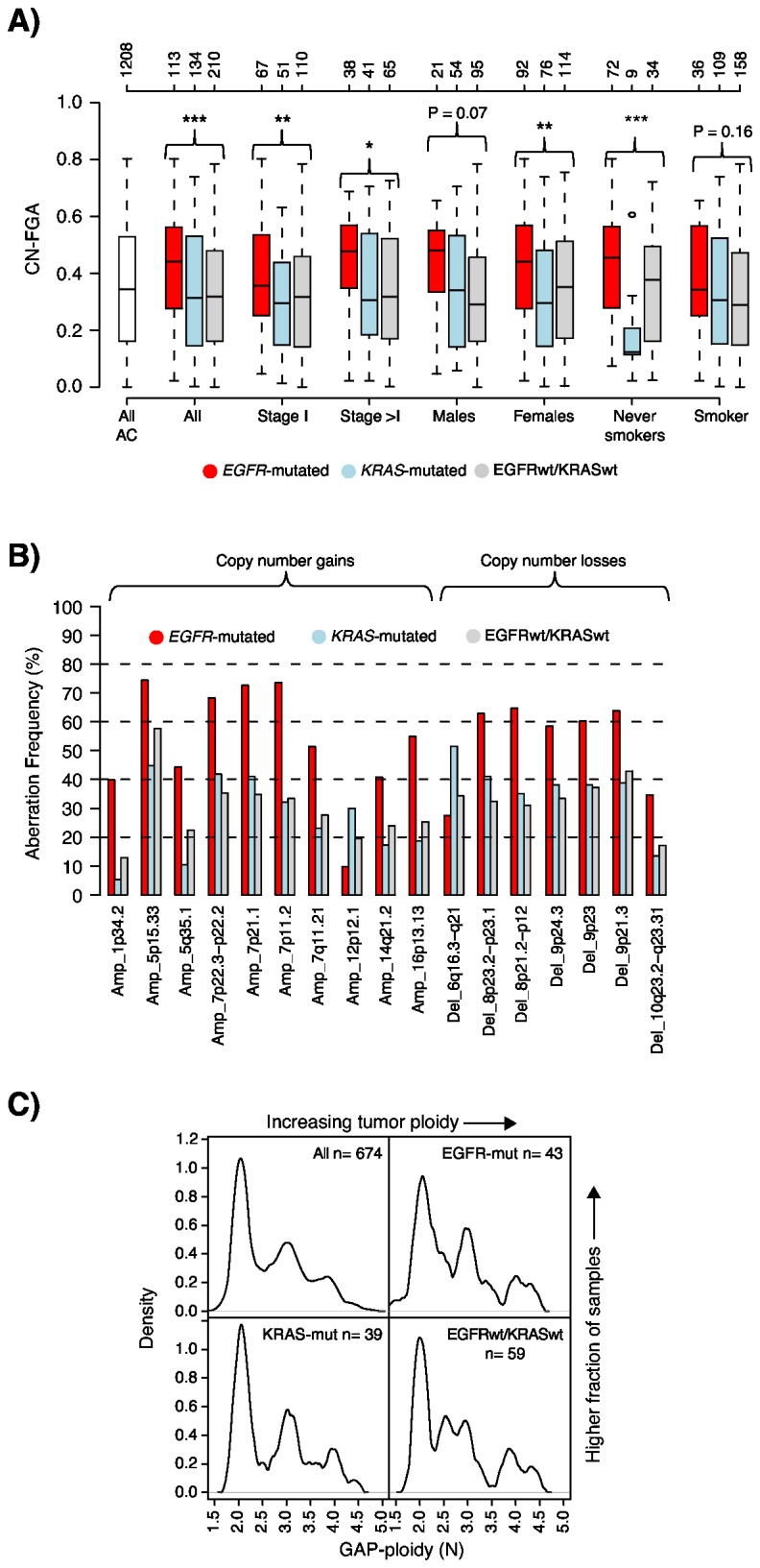
Copy number alterations and tumor ploidy in EGFR/KRAS mutation groups. (**A**) Pattern of gross copy number alterations measured as fraction of the genome altered by copy number gain or loss in adenocarcinoma tumors stratified by *EGFR* and *KRAS* mutation status (EGFR:red, KRAS:light blue, EGFRwt/KRASwt:gray), stage, gender and patient smoking status. Copy number alterations were called using log_2_ratio ± 0.12 as thresholds for identification of copy number gain and loss. P-values were calculated using ANOVA for indicated groups, ***: P< 0.001, **: P< 0.01, *: P< 0.05. Top axis indicates number of cases per group. (**B**) mGISTIC regions discriminating between *EGFR*-mutated (red), *KRAS*-mutated (light blue) and EGFRwt/KRASwt (gray) adenocarcinoma tumors. mGISTIC regions identified by Fisher’s exact test (Bonferroni adjusted p-value < 0.05) with an additional requirement of > 20% frequency difference between the lowest and highest groups. The y-axis describes the frequency of copy number gain or loss in respective group. (**C**) Distribution of GAP-ploidy across the adenocarcinoma EGFR/KRAS mutation groups for 141 tumors analyzed by GAP. A GAP-ploidy of two equals a diploid, three a triploid genome and four a tetraploid genome. Curves were generated by an Epanechnikov smoothing kernel with 0.1 smoothing bandwidth.

Stratification of the 457 adenocarcinoma tumors with known *EGFR* and *KRAS* mutation status into *EGFR*-mutated (n=113), *KRAS*-mutated (n=134) and EGFRwt/KRASwt (n=210) tumors revealed both common alterations across mutation groups, such as gains of chromosome 1q and 8q, and loss of 3p, and regions with apparently different prevalence between mutation groups, including gains on chromosome 7p (*EGFR*-mutated) and 16p (*EGFR*-mutated), and losses on 6q (*KRAS*-mutated) (Figures 2B-D). In general, *EGFR*-mutated tumors displayed more copy number alterations (estimated by the fraction of the genome altered by CNA, CN-FGA) than non-*EGFR*-mutated tumors (Figure 3A). This pattern was consistent also in five out of six individual cohorts that included both *EGFR*-mutated and non-*EGFR*-mutated tumors. When the three mutation groups were stratified by clinicopathological variables, *EGFR*-mutated tumors continued to display higher CN-FGA fractions in stage I tumors, female patients, and never-smokers (Figure 3A).

Analysis of the 90 focal mGISTIC regions (derived from analysis of the 1272 adenocarcinoma sample cohort) identified 17 regions discriminating between the three mutation groups. 15 of these 17 regions showed the highest alteration frequency in *EGFR*-mutated tumors, while the remaining two regions showed highest frequency in *KRAS*-mutated tumors (Bonferroni adjusted Fisher’s exact test p< 0.05 and frequency difference >20%, Figure 3B and Table 3). Specifically, *EGFR*-mutated tumors showed higher frequencies of copy number gain on chromosomes 1p34.2 (including *MYCL*), 5p15.33, 5q35.1, 7p22.3-p22.2, 7p21.1, 7p11.2 (including *EGFR*), 7q11.21, 14q21.2, and 16p13.13, and copy number loss in regions at 8p (including *DUSP4*), 9p (including *CDKN2A*), and 10q23.2-q23.31 (*PTEN*). *KRAS*-mutated tumors showed higher frequencies of gain on 12p12.1 (*KRAS*) and loss at 6q16.3-q21. 

**Table 3:** Differences and similarities in genomic alterations and allelic imbalances between EGFR/KRAS mutation groups.

**Investigated property**	***EGFR*-mutated**	***KRAS*-mutated**	**EGFRwt/KRASwt**
Fraction of the genome altered by copy number gain and loss	More ^a^	Less	Less
Fraction of the genome altered by LOH	More	Less	Less
Fraction of the genome altered by copy number neutral LOH or copy-neutral allelic imbalance	Less	Less	Less
Overall frequency of recurrent amplifications and genomic complexity	More	Less	Less
Amplifications differing between mutation groups (mGISTIC regions)	7p11.2 (*EGFR*), 12q14.2-q14.3, 12q15 (*MDM2*)		8p12 (*FGFR1*)
Copy number alterations differing between mutation groups (mGISTIC regions)	+1p34.2, +5p15.33, +5q35.1, +7p22.3-p22.2, +7p21.1, +7p11.2, +7q11.21, +14q21.2, +16p13.13, -8p23.2-p23.1, -8p21.2-p12, -9p24.3, -9p23, -9p21.3, -10q23.2-q23.31	+12p12.1 (*KRAS*), -6q16.3-q21	
Copy number alterations differing between mutation groups (genome-wide screen)	+1p36.33-p31.1, +5q34-q35.3, +7p22.3-p11.1, +7q11.21, +16p13.3-p12.1, +16p11.2-q11.1, +21q22.11-q22.3, -8p22-p11.21, -8q11.23		
Characteristic total LOH regions (≥50% frequency)^b^	8p, 9, 13q, 17p	19p	
Predominant tumor ploidy (GAP-ploidy)	2N (highest), 3N	2N (highest), 3N	2N (highest), 3N

a *Les*s indicates relatively lower estimates or frequencies between groups. *More* indicates relatively higher estimates or frequencies.

b Includes LOH caused by copy number loss as well as copy-neutral LOH.

A genome-wide analysis of differences in copy number frequency between the three mutation groups identified nine large coherent genomic regions (seven gains and two losses), all with higher alteration frequency in *EGFR*-mutated tumors. Regions were located on 1p, 5q, 7p, 7q, 8p, 8q, 16p and 21q, and involved 8% (7% gain, 1% loss) of the analyzed genome (Hochberg adjusted Fisher’s exact test p<0.01 and minimum frequency difference >20%, Tables 3 and S2). 

Similar to copy number gain and loss in general, *EGFR*-mutated tumors also displayed more recurrent amplifications in the 59 mGISTIC regions of gain compared with the non-*EGFR*-mutated tumors (p=0.004, Chi-square test). This finding was consistent also in patients with stage I disease (p=0.02, Chi-square test) or female gender (p=0.004, Chi-square test). In higher stage (≥II) tumors and in male patients the *EGFR*-mutated group also showed more recurrent amplifications, however not reaching statistical significance due to the lower number of tumors in these comparisons. In exploratory analysis, individual recurrent amplifications at 7p11.2 (*EGFR*), 8p12 (*WHSC1L1*, *FGFR1*), and 12q14-q15 (including *MDM2*) discriminated between mutation groups (p<0.05, Fisher’s exact test, Table 3). 

Taken together, these results suggest a higher genomic complexity in *EGFR*-mutated adenocarcinomas compared with *KRAS*-mutated and EGFRwt/KRASwt tumors.

### Patterns of tumor ploidy and allelic imbalance in EGFR/KRAS mutation groups

Patterns of tumor ploidy and allelic imbalances between mutational groups were evaluated by GAP [30] analysis of 141 tumors (n=43 *EGFR*-mutated, 39 *KRAS*-mutated, and 59 EGFRwt/KRASwt) profiled by SNP microarrays. Primarily, no differences in distribution of tumor ploidy (estimated by GAP-ploidy) were observed between mutation groups (p=0.96, ANOVA, Figure 3C). Secondly, *EGFR*-mutated adenocarcinomas were weakly associated with higher fractions of total LOH compared with *KRAS*-mutated and EGFRwt/KRASwt tumors both overall and in stage I disease (p=0.05, ANOVA), but not in tumors of higher stages (≥II), or tumors stratified by gender. In contrast, no significant differences in copy-neutral LOH or copy-neutral allelic imbalance fractions were observed between mutation groups overall or when sub-stratified by stage or gender. The highest frequencies of total LOH (>50%) were most often found in regions of copy number loss, while copy-neutral LOH and copy-neutral allelic imbalance showed an overall lower prevalence across chromosomes in all mutation groups (generally ≤10-15% frequency for copy-neutral LOH, and <25% for copy-neutral allelic imbalance across chromosomes) (Table 3 and Figure S2). 

Taken together, this implies that the weak associations of differences in allelic imbalances between the mutation groups are predominantly related to LOH caused by copy number loss in *EGFR*-mutated tumors. 

### Supervised and unsupervised analysis of transcriptional differences between EGFR/KRAS mutation groups

To identify a robust set of differentially expressed genes between the three mutation groups we performed supervised analysis of five adenocarcinoma Affymetrix cohorts (n=624 tumors, discovery cohorts, Table 2). 96 genes showed consistent differential expression in ≥4 cohorts, while only 21 genes were differentially expressed across all five cohorts (Tables 4 and S3). We validated the 96 identified genes in four independent adenocarcinoma cohorts analyzed by different microarray platforms (n=290 tumors, Table 2). In the independent cohorts, 41-96% of the 96 genes were present and thus available for further comparisons. Of the available genes 46-67% showed differential expression between the mutation groups in the independent cohorts (p<0.05 ANOVA, Table S3).

**Table 4:** Differentially expressed genes between *EGFR*/*KRAS* mutation groups in ≥4 of five Affymetrix adenocarcinoma cohorts.

Gene	Name	Gene	Name
ACSF2	acyl-CoA synthetase family member 2	ISG20	interferon stimulated exonuclease gene 20kDa
ADCY9	adenylate cyclase 9	ITPR3	inositol 1,4,5-triphosphate receptor, type 3
AGFG1	*ArfGAP with FG repeats 1*	KCNK5 *	potassium channel, subfamily K, member 5
AHR	aryl hydrocarbon receptor	KIAA0319L *	polycystic kidney disease 1-like
APOH	apolipoprotein H precursor	KIAA0494	hypothetical protein LOC9813
ARMCX6	armadillo repeat containing, X-linked 6	KIAA0495	hypothetical protein LOC57212
ARSD	arylsulfatase D	KIAA1033 *	hypothetical protein LOC23325
BAG1	BCL2-associated athanogene 1	KRAS	c-K-ras2 protein isoform b
BLVRA	biliverdin reductase A	LDLRAP1 *	low density lipoprotein receptor adaptor protein
C16orf58	hypothetical protein LOC64755	LRRC31	leucine rich repeat containing 31
C7orf23	chromosome 7 open reading frame 23	MANBA	mannosidase, beta A, lysosomal
CADPS2	Ca2+-dependent activator protein for secretion 2	MEAF6 *	MYST/Esa1-associated factor 6
CAMTA1	calmodulin-binding transcription activator 1	MMP15	matrix metalloproteinase 15 preproprotein
CLDN10	claudin 10	MTPAP	mitochondrial poly(A) polymerase
COL21A1	collagen, type XXI, alpha 1 precursor	MYST1	MYST histone acetyltransferase 1
CTNNBIP1	catenin, beta interacting protein 1	NAT15	N(alpha)-acetyltransferase 60, NatF catalytic subunit
DDAH1 *	dimethylarginine dimethylaminohydrolase 1	NBPF10	hypothetical protein LOC440673
DDX21	DEAD (Asp-Glu-Ala-Asp) box polypeptide 21	NFYC *	nuclear transcription factor Y, gamma
DNAJC9	DnaJ homolog, subfamily C, member 9	NIPAL3	NIPA-like domain containing 3
DUSP4 *	dual specificity phosphatase 4	PDSS1	prenyl diphosphate synthase, subunit 1
EFHC2	EF-hand domain (C-terminal) containing 2	PEF1	penta-EF-hand domain containing 1
EGFR *	epidermal growth factor receptor	PER3	period 3
ELN	elastin	PIGV *	phosphatidylinositol glycan class V
ENC1	ectodermal-neural cortex (with BTB-like domain)	PIK3IP1	HGFL protein
ENTPD4	ectonucleoside triphosphate diphosphohydrolase	PPCS	phosphopantothenoylcysteine synthetase isoform
ETV5	ets variant gene 5 (ets-related molecule)	PPFIBP2	PTPRF interacting protein, binding protein 2
FAAH	fatty acid amide hydrolase	PPIF	peptidylprolyl isomerase F precursor
FAM184A	family with sequence similarity 184, member A	PRDM4	PR domain containing 4
FGF13	fibroblast growth factor 13	PYROXD1	pyridine nucleotide-disulphide oxidoreductase domain 1
FGG	fibrinogen, gamma chain	RAPGEF5	Rap guanine nucleotide exchange factor (GEF) 5
FGGY	FGGY carbohydrate kinase domain containing	RFK	riboflavin kinase

**Figure 4:**
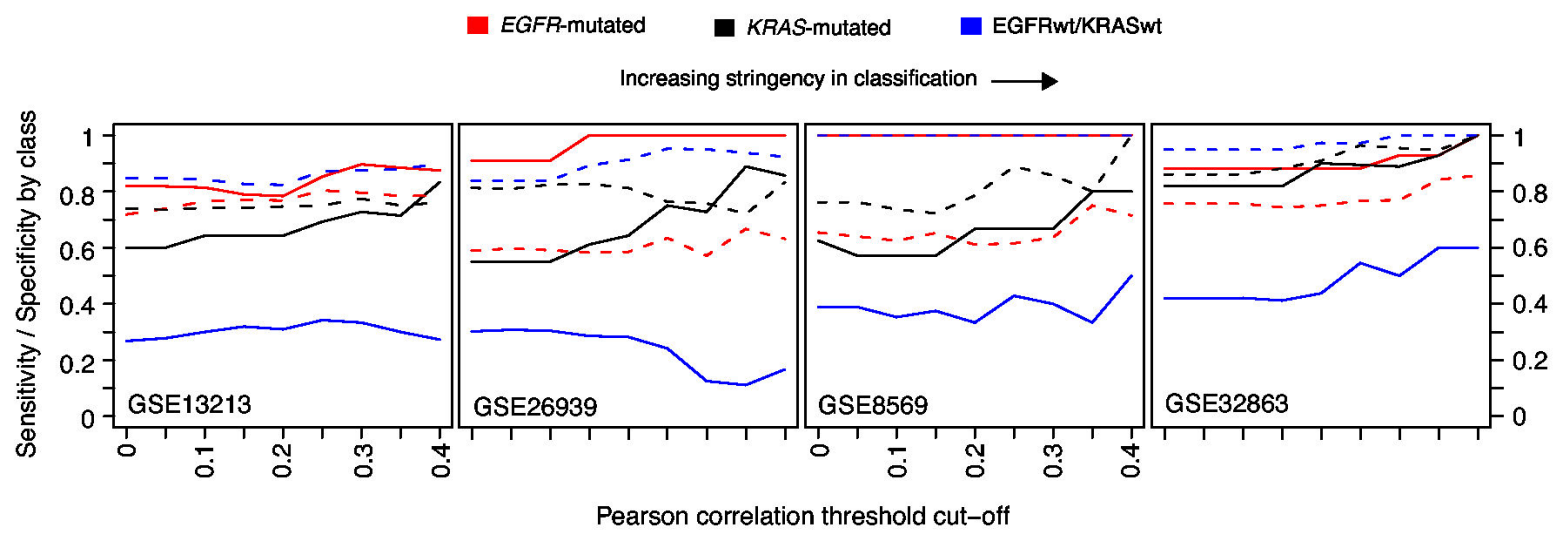
Supervised classification of adenocarcinoma gene expression cohorts with respect to *EGFR* and *KRAS* mutation status. Sensitivity (solid line) and specificity (dashed line) by EGFR/KRAS mutation group for classification of four independent validation cohorts using a 96-gene centroid classifier. The x-axis shows Pearson correlation cut-off for assigning a sample to the centroid with the highest correlation. Increasing correlation cut-offs correspond to increased stringency in classification, but introduces growing numbers of unclassified samples excluded in the calculation of sensitivity and specificity.

A three-group centroid classifier was used to explore the predictive power of the 96 genes in calling true mutation status (File S1 and Table S3). Classification of the four independent cohorts showed an overall accuracy of 40-90% in classification across a range of classification cut-offs (Figure S3A). Sensitivity was highest in classification of *EGFR*-mutated tumors across the different cohorts (80-100%), followed by *KRAS*-mutated tumors (Figure 4A). However, specificities of the 96-gene classifier were lower (60-90%) for the *EGFR* and *KRAS*-mutated groups (Figure 4A). In contrast, for EGFRwt/KRASwt tumors, sensitivity was poor (10-60%) but specificity higher (80-100%).

To further analyze the transcriptional patterns between the three mutation groups we performed unsupervised hierarchical clustering of four Affymetrix discovery cohorts (Table 2). Each cohort was individually clustered using a) a signature of genes overexpressed in EGFRwt/KRASwt tumors [26], b) a *KRAS* dependency gene signature [33], and c) probe sets derived from three different expression variance filters reflecting at different stringency the variation in expression across all tumors in a cohort (Affymetrix probe set range n=1356-24052). In none of these analyses did clustering resolve the three mutation groups into discrete transcriptional groups without notable inclusion of tumors from other mutation groups (Figure S4). However, supportive of results from the supervised analyses we found that *EGFR*-mutated adenocarcinomas in general appeared to display a more distinctive expression pattern with enrichment of *EGFR*-mutated tumors (~60% of all mutations) in specific clusters. In contrast, *KRAS*-mutated and EGFRwt/KRASwt tumors often appeared more intermixed, even when clustered using the *KRAS* dependency gene signature [33] (Figure S4). 

Taken together, results from the supervised and unsupervised gene expression analyses suggest that mutation status is not translated into a clearly distinctive and prominent expression signature.

## Discussion

In the current study we delineate genomic and transcriptional alterations in lung adenocarcinoma stratified by *EGFR* and *KRAS* mutation status. We show that a few specific copy number and transcriptional alterations exist between the three mutational groups, but also a considerable similarity caused by high intra-group heterogeneity and/or less distinctive inter-group differences. Together, this suggests that the overall genomic and transcriptional landscape of adenocarcinoma is affected, but only to a minor extent, by the mutational status of *EGFR* and *KRAS*.

Stratification of genomic profiles from 457 tumors with available *EGFR* and *KRAS* mutation status into three mutation groups revealed differences in the overall pattern of CNAs, amplifications and genomic architecture, as well as specific regions and amplifications differing in frequency between the groups (summarized in Table 3). Overall, *EGFR*-mutated tumors displayed more CNAs, more amplifications, and higher genomic complexity than non-*EGFR*-mutated tumors consistent with previous reports [11,15,18]. Specific patterns of recurrent amplifications in between the mutation groups, such as 8p12 (harboring *FGFR1*) in EGFRwt/KRASwt and 12q amplifications (including the p53 repressor *MDM2*) in *EGFR*-mutated tumors were observed. *FGFR1* mutations are rarely observed in NSCLC, while *FGFR1* amplification is frequent in, e.g., squamous cell lung carcinoma and associated with increased protein levels and a FGFR1 proliferation dependency [34]. Moreover, FGF-FGFR pathway activation has been suggested to be one mediator of resistance to EGFR inhibitors, together with, e.g., *MET* amplification (see [35] for review and [36]). In the current study, *FGFR1* and *MET* amplifications were restricted to the EGFRwt/KRASwt tumor group, and were mutually exclusive (*MET* amplification was borderline non-significant for difference in frequency between mutation groups, p=0.09, Fisher’s exact test). Together, this could indicate presence of specific genomic circuits acting as driving forces in pathogenesis in the different mutation groups. 

Taken together, the analyses of differential genomic regions point to only a few, variably sized, regions with moderate frequency differences (20-40%) between the mutational groups. These regions predominantly include regions of copy number gain with higher frequency in *EGFR*-mutated tumors. Several of the regions have been reported previously, but as larger and less defined regions [9,11-13,17,18], while others such as 5q34-q35.3, appear novel (see Table S4 for literature comparison of 34 previously reported regions from five independent studies [9,11-13,18]). For instance, 17 of our mGISTIC regions were present in 34 previously reported regions differing between *EGFR*-mutated and *EGFR*-wild type tumors, or *KRAS*-mutated tumors and *KRAS*-wild type tumors (Table S4). Although 24 of the 34 reported regions showed statistical significance for the original comparisons in our cohort, only 16 of these 24 regions also showed >20% frequency difference between the three groups. The absolute majority of these regions (88%) were located on chromosome 1p, 7p, and 16p (gains) and 8p (losses). Together, this emphasizes the need for adequately sized cohorts in order to draw reproducible conclusions when only moderate differences exist between investigated groups.

Few genome-wide analyses of differential allelic imbalance between *EGFR*-mutated, *KRAS*-mutated, and EGFRwt/KRASwt tumors exist in the literature. Blons et al. reported that *EGFR*-mutated tumors in general display more fractional allelic loss than *KRAS*-mutated tumors [18], consistent with our observation of higher fractions of total LOH in *EGFR*-mutated tumors. Moreover, Nakanishi et al. reported that two regions, 4q13 and 4q22, differ in allelic imbalance between the mutation groups [37]. However, in the current study we could not verify this finding using total LOH, copy-neutral LOH or copy-neutral allelic imbalance as measurements. Instead, we found that the frequency of total LOH was strongly correlated with regions of copy number loss. In contrast, the less frequent copy-neutral LOH and copy-neutral allelic imbalance events were overall more evenly distributed across chromosomes in the mutation groups. These findings are consistent with results for lung cancer histology groups in general [21], and also recent reports from breast cancer [38,39], suggesting that LOH is predominantly caused by copy number loss in these tumor types. Moreover, the similarity in the observed tumor ploidy patterns appears consistent with that the mutation groups do not exhibit gross differences in either CNAs or allelic imbalances. However, it should be noted that the analyses of allelic imbalances in the current study are based on a smaller subset of samples (n=141), which could be a source of variability.

Several studies have reported *KRAS* mutant signatures or differentially expressed genes between adenocarcinomas with *EGFR* and/or *KRAS* mutations and respective wild type cases [8,10,12,14,16,26,33,40]. However, the overlap between these public signatures is very poor when directly compared (Figures S3B and C). We identified 96 differentially expressed genes by supervised gene expression analyses between mutation groups across multiple discovery cohorts, of which several could be validated in independent cohorts. Reasons for the lower number of significant genes in the independent cohorts likely include smaller sample sizes and different microarray platforms compared with the discovery cohorts. The low number of differentially expressed genes between the mutation groups (only 21 genes consistently differentially expressed in all five discovery cohorts) is similar to results from other studies [8,12,14,16]. This low number of differentially expressed genes argues against that the mutational subgroups represent distinct transcriptional groups. Moreover, the overlaps between our 96 genes and previous studies [8,14,16,26,33,40] were poor (1-5% individual overlap between signatures). These results underline the need for a multicohort approach for identification of robust transcriptional differences between the mutation groups. Notably, our 96 genes mapped to a higher extent (43% of genes) to genomic regions showing differences in frequency of copy number gain or loss between the mutation groups compared to gene signatures from five reported studies [8,12,14,16,26] (6-15% of reported genes). However, the influence of the modest differences in CNA frequency (20-40%) between the mutation groups on transcriptional levels is difficult to assess. In addition to *EGFR* and *KRAS*, differentially expressed genes between the mutation groups included several other genes reported to be involved in tumorigenesis (*DUSP4*, *RPS6KA1*, *ID1*, *TNFRSF10B*, *CAMTA1*) [10,41], and, consistent with the enrichment of never-smokers in the *EGFR*-mutated patient group, genes reported as deregulated by smoking (*AHR*, *CLDN10*, *FGG*, *GGA2*, *GUSB*, *TXNRD1*) [42-45].

In supervised classification, the 96 differentially expressed genes identified *EGFR*-mutated adenocarcinomas with high sensitivity, but poorer specificity, while opposite results was found for EGFRwt/KRASwt tumors. Together with the results from unsupervised hierarchical clustering of multiple gene expression cohorts using different gene or probe sets these analyses demonstrate the difficulty in separating the mutation groups, especially *KRAS*-mutated and EGFRwt/KRASwt tumors, into more discrete transcriptional entities. Chitale et al. [10] proposed that the more distinctive expression pattern of *EGFR*-mutated tumors compared to *KRAS*-mutated tumors may depend on either a less prominent effect of *KRAS* mutations on expression, a biological or etiological heterogeneity among *KRAS*-mutated tumors, or that *EGFR* mutations arise in a more homogeneous and restricted cell type. Our results may be interpreted as support for potentially all three hypotheses, given the differences observed between and within mutation groups. Together, the results from our supervised and unsupervised gene expression analyses suggest that only modest, reproducible, transcriptional differences exist between the mutation groups. This conclusion appears consistent with the somewhat mixed inclusion of *EGFR*-mutated, *KRAS*-mutated and EGFRwt/KRASwt adenocarcinomas in different reported molecular subtypes of adenocarcinomas [16,19,20]. Although the bronchioid molecular subtype [19] has been strongly associated with *EGFR*-mutated tumors, this subtype also includes notable fractions of *KRAS*-mutated and EGFRwt/KRASwt tumors (see, e.g., [19,20]). Moreover, ~30% or more of *EGFR*-mutated have been classified as non-bronchioid (magnoid or squamoid) in discovery cohorts in previous studies [19,20,46]. In the absence of bronchioid classified tumors we found no significant association between the magnoid and squamoid subtypes and EGFR/KRAS mutation status in any of the five discovery cohorts in the current study (data not shown). These findings appear consistent with our unsupervised analysis showing a more distinct expression pattern of a subset of *EGFR*-mutated tumors across multiple cohorts, while the *KRAS*-mutated and EGFRwt/KRASwt groups are more intermixed (Figure S4). These results also suggest that *EGFR*-mutated tumors could be divided into additional subgroups, which we have recently demonstrated [46]. Taken together, *EGFR* and *KRAS* mutational status do not appear to be translated into a clearly distinctive and prominent expression signature in lung adenocarcinoma.

To further delineate the observed heterogeneous patterns of CNAs, allelic imbalances and gene expression patterns in the three mutational groups identification and/or definition of new molecular subgroups within the *EGFR*-mutated, *KRAS*-mutated and EGFRwt/KRASwt tumor groups are needed. For instance, although EGFRwt/KRASwt adenocarcinomas with *ALK* rearrangements are reported to display distinct expression profiles compared with *ALK*-negative tumors [26], it remains unclear whether this is also true for CNAs and allelic imbalances. Recent studies of lung adenocarcinoma have suggested that molecular profiling could be of value in future clinical decision making by providing clues about, e.g., treatment response to EGFR inhibitors [17,20,47,48]. For instance, Yuan et al. recently reported that clustered CNAs (copy number gains) on chromosome 7p were associated with poorer survival and less favorable response to EGFR tyrosine kinase inhibitors in *EGFR*-mutated adenocarcinomas specifically [17]. In support of Yuan et al., we recently identified a gene signature associated with poorer survival for patients with *EGFR*-mutated adenocarcinomas, where the high-risk patient group showed more copy number gains and amplifications on chromosome 7p [46]. As regions on chromosome 7p display some of the largest frequency differences between the mutational groups (~40%) these findings highlight the need for a more detailed characterization of this chromosome arm. The growing number of detected tyrosine kinase fusions in predominantly EGFRwt/KRASwt adenocarcinomas (including *ALK*, *RET*, and *ROS1*) are also becoming increasingly important in the therapeutic setting, as these alterations are/may become targets for specialized molecular agents. However, it remains to be investigated whether there exist similar regions and/or gene signatures associated with treatment response also for these adenocarcinoma subgroups. Clearly, further molecular stratification within the *EGFR* and *KRAS* mutation-defined lung adenocarcinoma groups has the potential to reveal new targets for synergistic treatment and provide insights into resistance mechanisms.

In summary, our multicohort analyses of genomic and transcriptional alterations demonstrate both differences and strong similarities between the *EGFR* and *KRAS* mutation defined adenocarcinoma groups. Moreover, our results suggest that the overall genomic and transcriptional landscape of adenocarcinoma is only to a minor extent affected by the mutational status of *EGFR* and *KRAS*.

## Supporting Information

Figure S1 **Permutation analysis of mGISTIC regions.** Close support from permutation analysis for an mGISTIC region (n=90) is defined as the % of times the region was enclosed or overlapped by a permuted region based on a 75% sample subset of the 1272 samples (n=100 permutations). (**A**) Cumulative fraction of regions (all, gain, loss) stratified into bins of 10% close support. (**B**) Hexagonal binning of mGISTIC regions (all, gain, loss) for % of close support versus -log_10_(p-value) of detected regions. A general trend of higher p-values connected to lower % close support is observed. Colors of bins indicate number of regions. Taken together, regions showing the lowest permutation detection rates also showed the lowest g-scores [28] and p-values. This is consistent with that these regions are present in only a small subset of the 1272 cases, which makes the identification and delineation of these regions to sensitive to sample composition.
(PDF)

Figure S2 **Pattern of CNAs, LOH, CNN-LOH, and CNN-AI in EGFR/KRAS mutation groups.** Panels show in decreasing order from the top pattern (frequency) of copy number gain (red) and loss (green) relative to GAP-ploidy with mGISTIC regions identified from the 1272 sample cohort indicated by blue dots, LOH, copy-neutral LOH (CNN-LOH), copy-neutral allelic imbalance (CNN-AI), and variation of FGA values versus GAP-ploidy for copy number (black), CNN-AI (red), LOH (blue), and CNN-LOH (light blue) in the bottom panel. For the bottom panel GAP-ploidy estimates were binned in bins of size 0.3, which is represented by tick marks on the x-axis. For each bin the median FGA value of the included samples is plotted (points) for copy number, LOH, CNN-LOH and CNN-AI. Bins contain different numbers of samples (top axis). The 141 tumors with mutation status analyzed by GAP were stratified into (**A**) *EGFR*-mutated (n=43), (**B**) *KRAS*-mutated (n=39), and (**C**) EGFRwt/KRASwt (n=59) tumors.
(PDF)

Figure S3 **Comparison of public EGFR/KRAS signatures and classification by a set of genes differentially expressed between EGFR/KRAS mutation groups across multiple cohorts.** (**A**) Overall accuracy for classification of four independent adenocarcinoma cohorts using a 96-gene centroid classifier. The number of genes in the centroid matching to the different cohorts varies. The x-axis shows Pearson correlation cut-off for assigning a sample to the centroid with the highest correlation. Increasing correlation cut-offs introduces growing numbers of unclassified samples, which are excluded in calculation of accuracy. (**B**) Venn-diagram of the gene overlap between four reported gene lists of differentially expressed genes between *EGFR*-mutated and *EGFR*-wild type adenocarcinoma tumors. (**C**) Venn-diagram of the gene overlap between two reported gene lists of differentially expressed genes between *KRAS*-mutated and *KRAS*-wild type adenocarcinoma tumors, and two reported *KRAS* mutant signatures [33,40].
(PDF)

Figure S4 **Unsupervised analyses of four Affymetrix adenocarcinoma gene expression cohorts using different probe sets.** Unsupervised hierarchical clustering was performed using Pearson correlation and complete linkage using five different probe sets in four adenocarcinoma cohorts that were analyzed by Affymetrix gene expression microarrays. Dendrograms for each cluster tree were cut into the top two or three clusters, and the number of probe sets used in the clustering is shown for each cohort. For each cohort the distribution of *EGFR*-mutated (red), *KRAS*-mutated (blue), and EGFRwt/KRASwt (black) tumors are shown across clusters as bars. Percentages in bar plots correspond to, e.g., how many *EGFR*-mutated tumors of the total number of *EGFR*-mutated cases that reside in a particular cluster. (**A**) Clustering based on probe sets from a list of 190 probe sets reported to be upregulated in EGFRwt/KRASwt adenocarcinomas [26]. (**B**) Clustering based on probe sets with log2ratio standard deviation >0.3 across tumors in a cohort. (**C**) Clustering based on probe sets with log2ratio standard deviation >0.5 across tumors in a cohort. (**D**) Clustering based on probe sets with log2ratio standard deviation >1 across tumors in a cohort. (**E**) Clustering based on matching genes from the list of top 250 genes reported by Singh et al. [33]. Each dendrogram is cut into the top two clusters. Division of dendrograms into three groups did not identify *KRAS*-mutants as a single group without notable inclusion of EGFRwt/KRASwt tumors in all cohorts.
(PDF)

File S1 **Document with details concerning used analysis methods.**
(DOC)

Table S1 **Genomic mGISTIC regions identified from analysis of 1272 lung adenocarcinomas.**
(XLSX)

Table S2 **Differential regions of copy number gain and loss between mutation groups obtained from genome-wide analysis.**
(XLSX)

Table S3 **Differentially expressed genes between mutation groups across at least four gene expression cohorts.**
(XLSX)

Table S4 **Analysis of genomic regions reported in the literature to stratify mutation groups in the current cohort.**
(DOC)

## References

[1] Jemal A, Bray F, Center MM, Ferlay J, Ward E et al. (2011) Global cancer statistics. CA Cancer J Clin 61: 69-90. doi:10.3322/caac.20107. PubMed: 21296855. 21296855

[2] Travis WD, Brambilla E, Muller-Hermelink HK, Harris CC (Eds.) (2004) World Health Organization Classification of Tumours. Pathology and Genetics of Tumours of the Lung, Pleura, Thymus and Heart. Lyon: IARC Press.

[3] Lynch TJ, Bell DW, Sordella R, Gurubhagavatula S, Okimoto RA et al. (2004) Activating mutations in the epidermal growth factor receptor underlying responsiveness of non-small-cell lung cancer to gefitinib. N Engl J Med 350: 2129-2139. doi:10.1056/NEJMoa040938. PubMed: 15118073. 15118073

[4] Pao W, Chmielecki J (2010) Rational, biologically based treatment of EGFR-mutant non-small-cell lung cancer. Nat Rev Cancer 10: 760-774. doi:10.1038/nrc2947. PubMed: 20966921. 20966921 PMC3072803

[5] Kadara H, Kabbout M, Wistuba II (2011) Pulmonary adenocarcinoma: a renewed entity in 2011. Respirology 17: 50-65. PubMed: 22040022. 10.1111/j.1440-1843.2011.02095.x PMC3911779 22040022

[6] Takeuchi K, Soda M, Togashi Y, Suzuki R, Sakata S et al. (2012) RET, ROS1 and ALK fusions in lung cancer. Nat Med 18: 378-381. doi:10.1038/nm.2658. PubMed: 22327623. 22327623

[7] Kwak EL, Bang YJ, Camidge DR, Shaw AT, Solomon B et al. (2010) Anaplastic lymphoma kinase inhibition in non-small-cell lung cancer. N Engl J Med 363: 1693-1703. doi:10.1056/NEJMoa1006448. PubMed: 20979469. 20979469 PMC3014291

[8] Angulo B, Suarez-Gauthier A, Lopez-Rios F, Medina PP, Conde E et al. (2008) Expression signatures in lung cancer reveal a profile for EGFR-mutant tumours and identify selective PIK3CA overexpression by gene amplification. J Pathol 214: 347-356. doi:10.1002/path.2267. PubMed: 17992665. 17992665

[9] Broët P, Dalmasso C, Tan EH, Alifano M, Zhang S et al. (2011) Genomic profiles specific to patient ethnicity in lung adenocarcinoma. Clin Cancer Res 17: 3542-3550. doi:10.1158/1078-0432.CCR-10-2185. PubMed: 21521776. 21521776

[10] Chitale D, Gong Y, Taylor BS, Broderick S, Brennan C et al. (2009) An integrated genomic analysis of lung cancer reveals loss of DUSP4 in EGFR-mutant tumors. Oncogene 28: 2773-2783. doi:10.1038/onc.2009.135. PubMed: 19525976. 19525976 PMC2722688

[11] Fong Y, Lin YS, Liou CP, Li CF, Tzeng CC (2010) Chromosomal imbalances in lung adenocarcinomas with or without mutations in the epidermal growth factor receptor gene. Respirology 15: 700-705. doi:10.1111/j.1440-1843.2010.01746.x. PubMed: 20409020. 20409020

[12] Newnham GM, Conron M, McLachlan S, Dobrovic A, Do H et al. (2011) Integrated mutation, copy number and expression profiling in resectable non-small cell lung cancer. BMC Cancer 11: 93. doi:10.1186/1471-2407-11-93. PubMed: 21385341. 21385341 PMC3058106

[13] Reinmuth N, Jauch A, Xu EC, Muley T, Granzow M et al. (2008) Correlation of EGFR mutations with chromosomal alterations and expression of EGFR, ErbB3 and VEGF in tumor samples of lung adenocarcinoma patients. Lung Cancer 62: 193-201. doi:10.1016/j.lungcan.2008.03.011. PubMed: 18450321. 18450321

[14] Shibata T, Hanada S, Kokubu A, Matsuno Y, Asamura H et al. (2007) Gene expression profiling of epidermal growth factor receptor/KRAS pathway activation in lung adenocarcinoma. Cancer Sci 98: 985-991. doi:10.1111/j.1349-7006.2007.00483.x. PubMed: 17459062. 17459062 PMC11159808

[15] Shibata T, Uryu S, Kokubu A, Hosoda F, Ohki M et al. (2005) Genetic classification of lung adenocarcinoma based on array-based comparative genomic hybridization analysis: its association with clinicopathologic features. Clin Cancer Res 11: 6177-6185. doi:10.1158/1078-0432.CCR-05-0293. PubMed: 16144918. 16144918

[16] Takeuchi T, Tomida S, Yatabe Y, Kosaka T, Osada H et al. (2006) Expression profile-defined classification of lung adenocarcinoma shows close relationship with underlying major genetic changes and clinicopathologic behaviors. J Clin Oncol 24: 1679-1688. doi:10.1200/JCO.2005.03.8224. PubMed: 16549822. 16549822

[17] Yuan S, Yu SL, Chen HY, Hsu YC, Su KY et al. (2011) Clustered genomic alterations in chromosome 7p dictate outcomes and targeted treatment responses of lung adenocarcinoma with EGFR-activating mutations. J Clin Oncol 29: 3435-3442. doi:10.1200/JCO.2011.35.3979. PubMed: 21810691. 21810691

[18] Blons H, Pallier K, Le Corre D, Danel C, Tremblay-Gravel M et al. (2008) Genome wide SNP comparative analysis between EGFR and KRAS mutated NSCLC and characterization of two models of oncogenic cooperation in non-small cell lung carcinoma. BMC Med Genomics 1: 25. doi:10.1186/1755-8794-1-25. PubMed: 18549475. 18549475 PMC2527324

[19] Hayes DN, Monti S, Parmigiani G, Gilks CB, Naoki K et al. (2006) Gene expression profiling reveals reproducible human lung adenocarcinoma subtypes in multiple independent patient cohorts. J Clin Oncol 24: 5079-5090. doi:10.1200/JCO.2005.05.1748. PubMed: 17075127. 17075127

[20] Wilkerson MD, Yin X, Walter V, Zhao N, Cabanski CR et al. (2012) Differential pathogenesis of lung adenocarcinoma subtypes involving sequence mutations, copy number, chromosomal instability, and methylation. PLOS ONE 7: e36530. doi:10.1371/journal.pone.0036530. PubMed: 22590557. 22590557 PMC3349715

[21] Staaf J, Isaksson S, Karlsson A, Jönsson M, Johansson L et al. (2012) Landscape of somatic allelic imbalances and copy number alterations in human lung carcinoma. Int J Cancer 1: 2020-2031. PubMed: 23023297. 10.1002/ijc.27879 23023297

[22] Tomida S, Takeuchi T, Shimada Y, Arima C, Matsuo K et al. (2009) Relapse-related molecular signature in lung adenocarcinomas identifies patients with dismal prognosis. J Clin Oncol 27: 2793-2799. doi:10.1200/JCO.2008.19.7053. PubMed: 19414676. 19414676

[23] Selamat SA, Chung BS, Girard L, Zhang W, Zhang Y et al. (2012) Genome-scale analysis of DNA methylation in lung adenocarcinoma and integration with mRNA expression. Genome Res 22: 1197-1211. doi:10.1101/gr.132662.111. PubMed: 22613842. 22613842 PMC3396362

[24] Fouret R, Laffaire J, Hofman P, Beau-Faller M, Mazieres J et al. (2012) A comparative and integrative approach identifies ATPase family, AAA domain containing 2 as a likely driver of cell proliferation in lung adenocarcinoma. Clin Cancer Res 18: 5606-5616. doi:10.1158/1078-0432.CCR-12-0505. PubMed: 22914773. 22914773

[25] Botling J, Edlund K, Lohr M, Hellwig B, Holmberg L et al. (2012) Biomarker discovery in non-small cell lung cancer: integrating gene expression profiling, meta-analysis and tissue microarray validation. Clin Cancer Res 19: 194-204. PubMed: 23032747. 23032747 10.1158/1078-0432.CCR-12-1139

[26] Okayama H, Kohno T, Ishii Y, Shimada Y, Shiraishi K et al. (2012) Identification of genes upregulated in ALK-positive and EGFR/KRAS/ALK-negative lung adenocarcinomas. Cancer Res 72: 100-111. doi:10.1158/1538-7445.AM2012-100. PubMed: 22080568. 22080568

[27] Micke P, Edlund K, Holmberg L, Kultima HG, Mansouri L et al. (2011) Gene copy number aberrations are associated with survival in histologic subgroups of non-small cell lung cancer. J Thorac Oncol 6: 1833-1840. doi:10.1097/JTO.0b013e3182295917. PubMed: 22011649. 22011649

[28] Mermel CH, Schumacher SE, Hill B, Meyerson ML, Beroukhim R et al. (2011) GISTIC2.0 facilitates sensitive and confident localization of the targets of focal somatic copy-number alteration in human cancers. Genome Biol 12: R41. doi:10.1186/1465-6906-12-S1-P41. PubMed: 21527027. 21527027 PMC3218867

[29] Staaf J, Lindgren D, Vallon-Christersson J, Isaksson A, Göransson H et al. (2008) Segmentation-based detection of allelic imbalance and loss-of-heterozygosity in cancer cells using whole genome SNP arrays. Genome Biol 9: R136. doi:10.1186/gb-2008-9-9-r136. PubMed: 18796136. 18796136 PMC2592714

[30] Popova T, Manié E, Stoppa-Lyonnet D, Rigaill G, Barillot E et al. (2009) Genome Alteration Print (GAP): a tool to visualize and mine complex cancer genomic profiles obtained by SNP arrays. Genome Biol 10: R128. doi:10.1186/gb-2009-10-11-r128. PubMed: 19903341. 19903341 PMC2810663

[31] Bolstad BM, Irizarry RA, Astrand M, Speed TP (2003) A comparison of normalization methods for high density oligonucleotide array data based on variance and bias. Bioinformatics 19: 185-193. doi:10.1093/bioinformatics/19.2.185. PubMed: 12538238. 12538238

[32] Gene Expression Omnibus. Available: http://www.ncbi.nlm.nih.gov/geo/.

[33] Singh A, Greninger P, Rhodes D, Koopman L, Violette S et al. (2009) A gene expression signature associated with "K-Ras addiction" reveals regulators of EMT and tumor cell survival. Cancer Cell 15: 489-500. doi:10.1016/j.ccr.2009.03.022. PubMed: 19477428. 19477428 PMC2743093

[34] Weiss J, Sos ML, Seidel D, Peifer M, Zander T et al. (2010) Frequent and focal FGFR1 amplification associates with therapeutically tractable FGFR1 dependency in squamous cell lung cancer. Sci Transl Med 2: 62ra93 PubMed: 21160078. 10.1126/scitranslmed.3001451 PMC3990281 21160078

[35] Kono SA, Marshall ME, Ware KE, Heasley LE (2009) The fibroblast growth factor receptor signaling pathway as a mediator of intrinsic resistance to EGFR-specific tyrosine kinase inhibitors in non-small cell lung cancer. Drug Resist Update 12: 95-102. doi:10.1016/j.drup.2009.05.001. PubMed: 19501013. PMC2763047 19501013

[36] Terai H, Soejima K, Yasuda H, Nakayama S, Hamamoto J et al. (2013) Activation of the FGF2-FGFR1 Autocrine Pathway: A Novel Mechanism of Acquired Resistance to Gefitinib in NSCLC. Mol Cancer Res 11: 759-767. doi:10.1158/1541-7786.MCR-12-0652. PubMed: 23536707. 23536707

[37] Nakanishi H, Matsumoto S, Iwakawa R, Kohno T, Suzuki K et al. (2009) Whole genome comparison of allelic imbalance between noninvasive and invasive small-sized lung adenocarcinomas. Cancer Res 69: 1615-1623. doi:10.1158/0008-5472.CAN-08-3218. PubMed: 19190329. 19190329

[38] Staaf J, Jönsson G, Ringnér M, Baldetorp B, Borg A (2011) Landscape of somatic allelic imbalances and copy number alterations in HER2-amplified breast cancer. Breast Cancer Res 13: R129. doi:10.1186/bcr3075. PubMed: 22169037. 22169037 PMC3326571

[39] Ha G, Roth A, Lai D, Bashashati A, Ding J et al. (2012) Integrative analysis of genome-wide loss of heterozygosity and monoallelic expression at nucleotide resolution reveals disrupted pathways in triple-negative breast cancer. Genome Res 22: 1995-2007. doi:10.1101/gr.137570.112. PubMed: 22637570. 22637570 PMC3460194

[40] Sweet-Cordero A, Mukherjee S, Subramanian A, You H, Roix JJ et al. (2005) An oncogenic KRAS2 expression signature identified by cross-species gene-expression analysis. Nat Genet 37: 48-55. PubMed: 15608639. 15608639 10.1038/ng1490

[41] Lara R, Mauri FA, Taylor H, Derua R, Shia A et al. (2011) An siRNA screen identifies RSK1 as a key modulator of lung cancer metastasis. Oncogene 30: 3513-3521. doi:10.1038/onc.2011.61. PubMed: 21423205. 21423205

[42] Beane J, Sebastiani P, Liu G, Brody JS, Lenburg ME et al. (2007) Reversible and permanent effects of tobacco smoke exposure on airway epithelial gene expression. Genome Biol 8: R201. doi:10.1186/gb-2007-8-9-r201. PubMed: 17894889. 17894889 PMC2375039

[43] Kitamura M, Kasai A (2007) Cigarette smoke as a trigger for the dioxin receptor-mediated signaling pathway. Cancer Lett 252: 184-194. doi:10.1016/j.canlet.2006.11.015. PubMed: 17189671. 17189671

[44] Landi MT, Dracheva T, Rotunno M, Figueroa JD, Liu H et al. (2008) Gene expression signature of cigarette smoking and its role in lung adenocarcinoma development and survival. PLOS ONE 3: e1651. doi:10.1371/journal.pone.0001651. PubMed: 18297132. 18297132 PMC2249927

[45] Bossé Y, Postma DS, Sin DD, Lamontagne M, Couture C et al. (2012) Molecular signature of smoking in human lung tissues. Cancer Res 72: 3753-3763. doi:10.1158/0008-5472.CAN-12-1160. PubMed: 22659451. 22659451

[46] Planck M, Isaksson S, Veerla S, Staaf J (2013) Identification of transcriptional subgroups in EGFR-mutated and EGFR/KRAS-wild type lung adenocarcinoma reveals gene signatures associated with patient outcome. Clin Cancer Res: [Epub ahead of print]. 10.1158/1078-0432.CCR-13-0928 23938291

[47] Yauch RL, Januario T, Eberhard DA, Cavet G, Zhu W et al. (2005) Epithelial versus mesenchymal phenotype determines in vitro sensitivity and predicts clinical activity of erlotinib in lung cancer patients. Clin Cancer Res 11: 8686-8698. doi:10.1158/1078-0432.CCR-05-1492. PubMed: 16361555. 16361555

[48] Byers LA, Diao L, Wang J, Saintigny P, Girard L et al. (2013) An epithelial-mesenchymal transition gene signature predicts resistance to EGFR and PI3K inhibitors and identifies Axl as a therapeutic target for overcoming EGFR inhibitor resistance. Clin Cancer Res 19: 279-290. doi:10.1158/1078-0432.CCR-12-1558. PubMed: 23091115. 23091115 PMC3567921

[49] Zhao X, Weir BA, LaFramboise T, Lin M, Beroukhim R et al. (2005) Homozygous deletions and chromosome amplifications in human lung carcinomas revealed by single nucleotide polymorphism array analysis. Cancer Res 65: 5561-5570. doi:10.1158/0008-5472.CAN-04-4603. PubMed: 15994928. 15994928

[50] Jaiswal BS, Janakiraman V, Kljavin NM, Chaudhuri S, Stern HM et al. (2009) Somatic mutations in p85alpha promote tumorigenesis through class IA PI3K activation. Cancer Cell 16: 463-474. doi:10.1016/j.ccr.2009.10.016. PubMed: 19962665. 19962665 PMC2804903

[51] Weir BA, Woo MS, Getz G, Perner S, Ding L et al. (2007) Characterizing the cancer genome in lung adenocarcinoma. Nature 450: 893-898. doi:10.1038/nature06358. PubMed: 17982442. 17982442 PMC2538683

[52] Beroukhim R, Mermel CH, Porter D, Wei G, Raychaudhuri S et al. (2010) The landscape of somatic copy-number alteration across human cancers. Nature 463: 899-905. doi:10.1038/nature08822. PubMed: 20164920. 20164920 PMC2826709

[53] Sos ML, Michel K, Zander T, Weiss J, Frommolt P et al. (2009) Predicting drug susceptibility of non-small cell lung cancers based on genetic lesions. J Clin Invest 119: 1727-1740. doi:10.1172/JCI37127. PubMed: 19451690. 19451690 PMC2689116

[54] Huang YT, Lin X, Chirieac LR, McGovern R, Wain JC et al. (2011) Impact on disease development, genomic location and biological function of copy number alterations in non-small cell lung cancer. PLOS ONE 6: e22961. doi:10.1371/journal.pone.0022961. PubMed: 21829676. 21829676 PMC3149069

[55] GlaxoSmith Kline Cancer Cell Line Genomic Profiling Data. Accessed 2013 9/23. Available: https://cabig.nci.nih.gov/tools/caArray_GSKdata.

[56] Lu TP, Tsai MH, Lee JM, Hsu CP, Chen PC et al. (2010) Identification of a novel biomarker, SEMA5A, for non-small cell lung carcinoma in nonsmoking women. Cancer Epidemiol Biomarkers Prev 19: 2590-2597. doi:10.1158/1055-9965.EPI-10-0332. PubMed: 20802022. 20802022

[57] The Cancer Genome Atlas. Available: http://cancergenome.nih.gov/.

[58] Kan Z, Jaiswal BS, Stinson J, Janakiraman V, Bhatt D et al. (2010) Diverse somatic mutation patterns and pathway alterations in human cancers. Nature 466: 869-873. doi:10.1038/nature09208. PubMed: 20668451. 20668451

[59] Job B, Bernheim A, Beau-Faller M, Camilleri-Broët S, Girard P et al. (2010) Genomic aberrations in lung adenocarcinoma in never smokers. PLOS ONE 5: e15145. doi:10.1371/journal.pone.0015145. PubMed: 21151896. 21151896 PMC2997777

