# Supplementary figures and images for: Genomic and Transcriptional Alterations in Lung Adenocarcinoma in Relation to EGFR and KRAS Mutation Status
Source: PLoS One. 2013 Oct 24;8(10):e78614. doi: 10.1371/journal.pone.0078614 (PMC3812039; doi:10.1371/journal.pone.0078614)

**A)**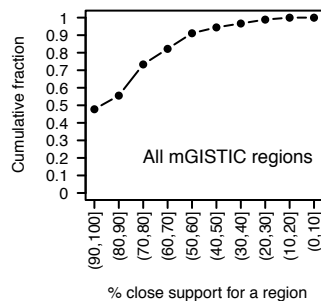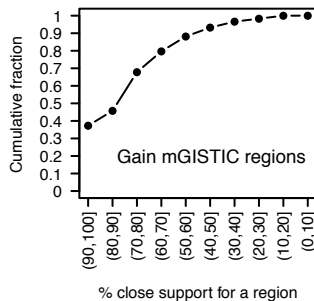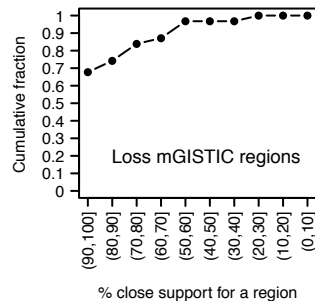**B)**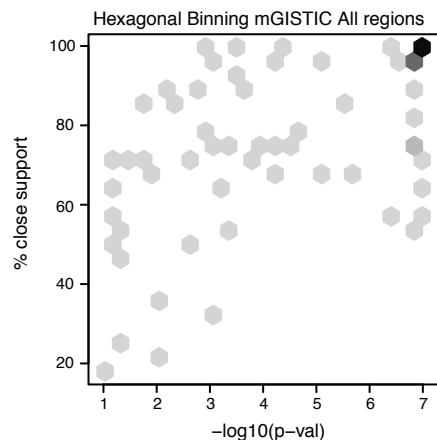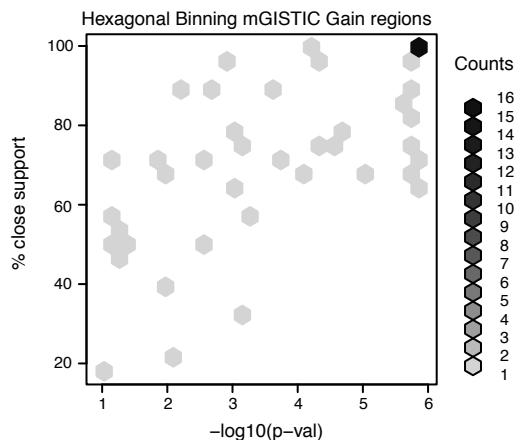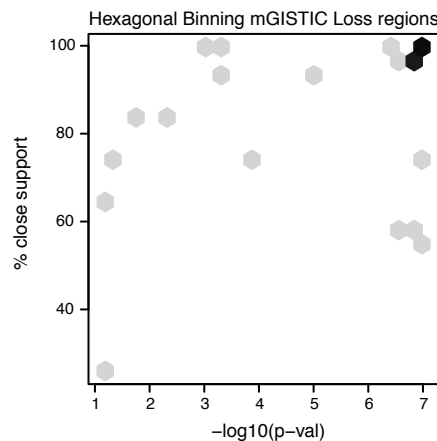

Supplement: Figure S1 — Permutation analysis of mGISTIC regions. Close support from permutation analysis for an mGISTIC region (n=90) is defined as the % of times the region was enclosed or overlapped by a permuted region based on a 75% sample subset of the 1272 samples (n=100 permutations). (A) Cumulative fraction of regions (all, gain, loss) stratified into bins of 10% close support. (B) Hexagonal binning of mGISTIC regions (all, gain, loss) for % of close support versus -log10(p-value) of detected regions. A general trend of higher p-values connected to lower % close support is observed. Colors of bins indicate number of regions. Taken together, regions showing the lowest permutation detection rates also showed the lowest g-scores [28] and p-values. This is consistent with that these regions are present in only a small subset of the 1272 cases, which makes the identification and delineation of these regions to sensitive to sample composition. (PDF) [file pone.0078614.s001.pdf]

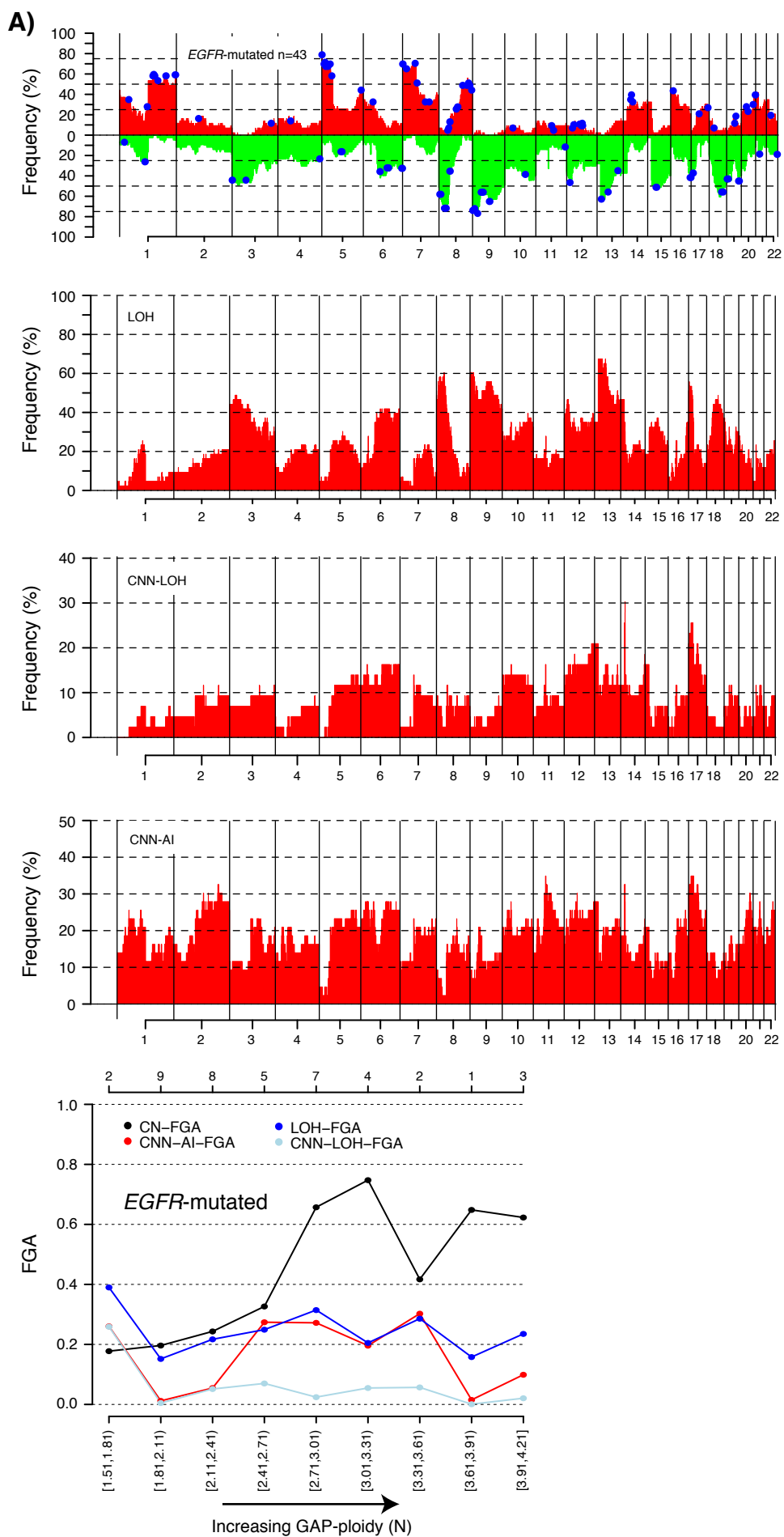

**B)**

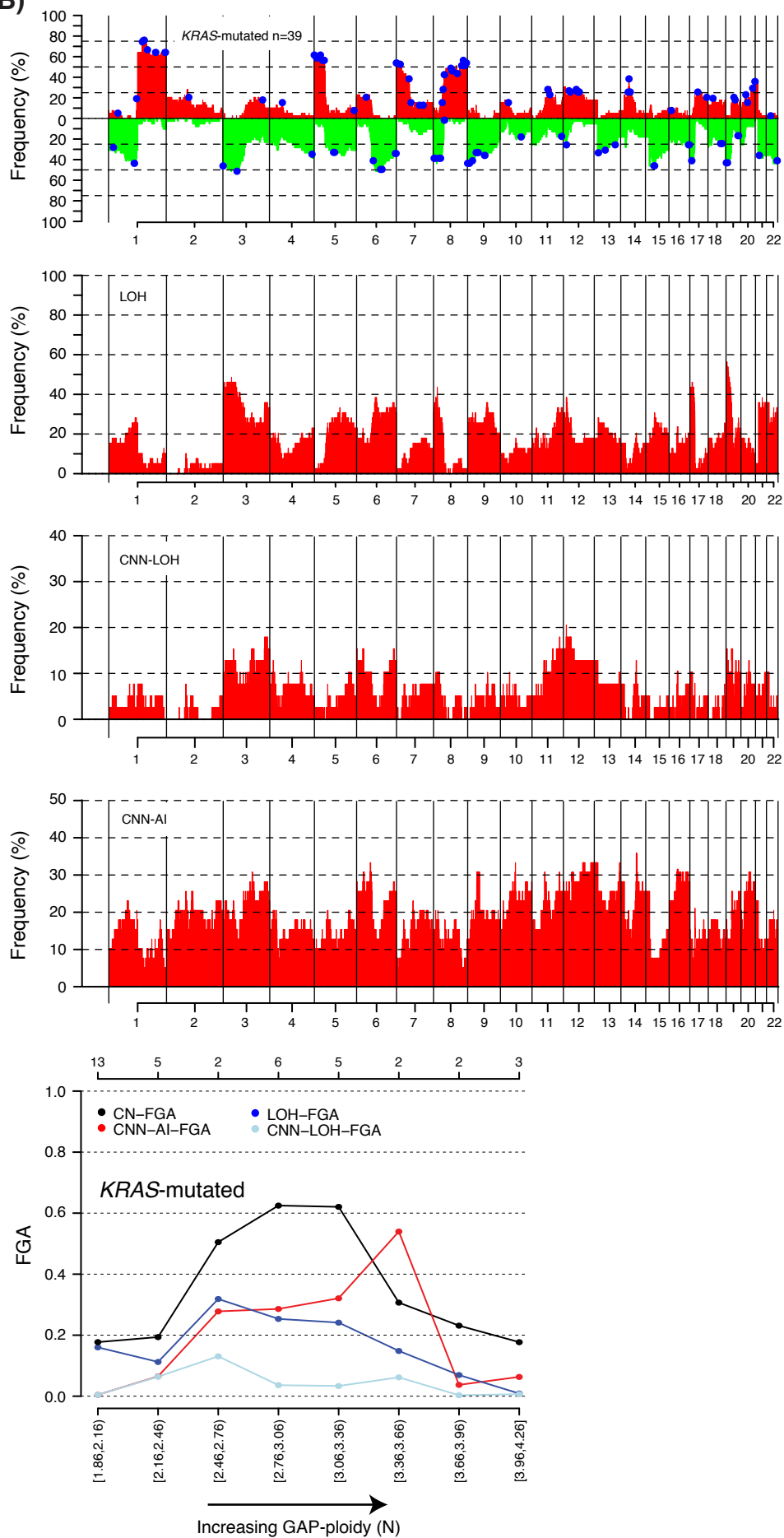

c)

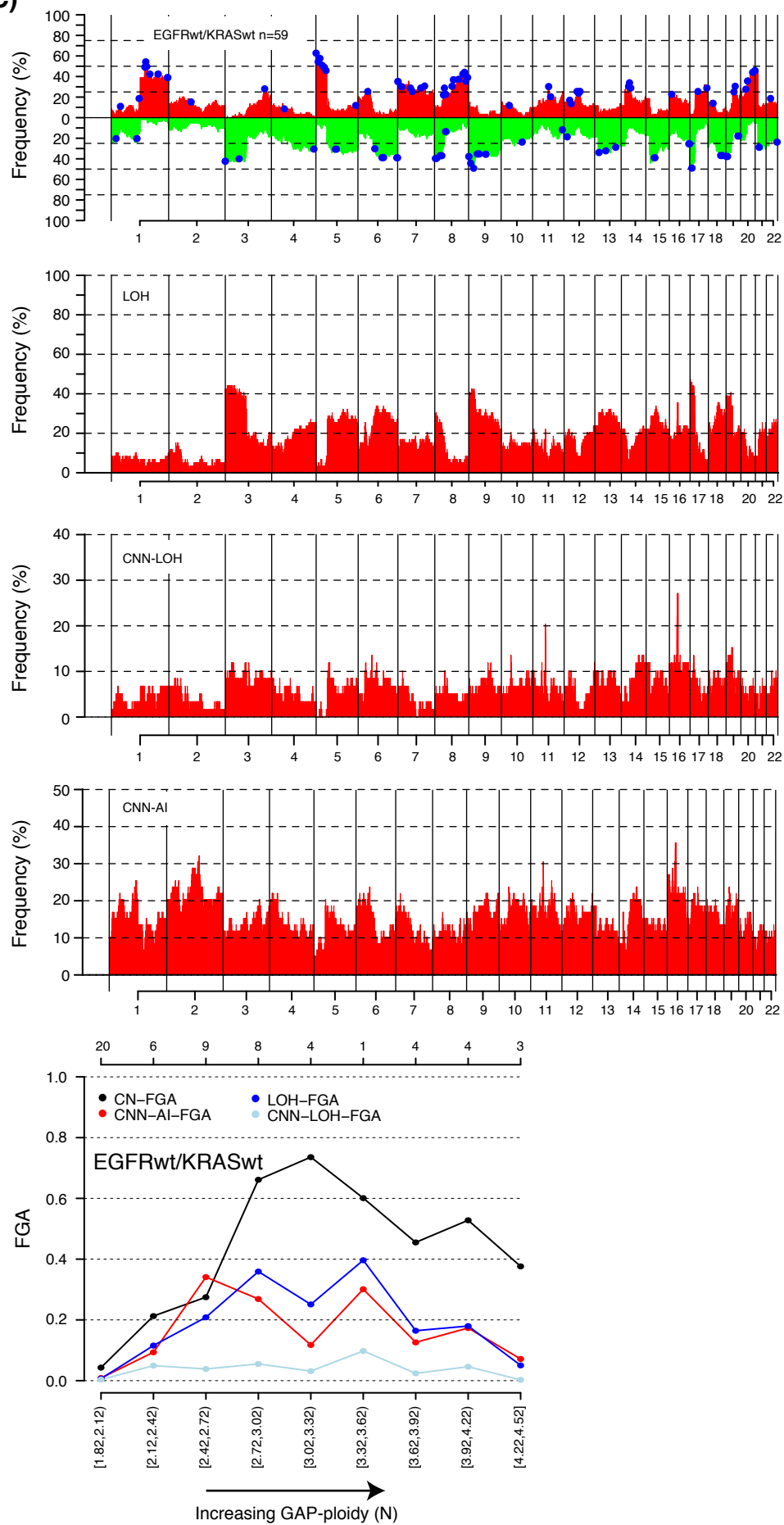

Supplement: Figure S2 — Pattern of CNAs, LOH, CNN-LOH, and CNN-AI in EGFR/KRAS mutation groups. Panels show in decreasing order from the top pattern (frequency) of copy number gain (red) and loss (green) relative to GAP-ploidy with mGISTIC regions identified from the 1272 sample cohort indicated by blue dots, LOH, copy-neutral LOH (CNN-LOH), copy-neutral allelic imbalance (CNN-AI), and variation of FGA values versus GAP-ploidy for copy number (black), CNN-AI (red), LOH (blue), and CNN-LOH (light blue) in the bottom panel. For the bottom panel GAP-ploidy estimates were binned in bins of size 0.3, which is represented by tick marks on the x-axis. For each bin the median FGA value of the included samples is plotted (points) for copy number, LOH, CNN-LOH and CNN-AI. Bins contain different numbers of samples (top axis). The 141 tumors with mutation status analyzed by GAP were stratified into (A) EGFR-mutated (n=43), (B) KRAS-mutated (n=39), and (C) EGFRwt/KRASwt (n=59) tumors. (PDF) [file pone.0078614.s002.pdf]

**A)**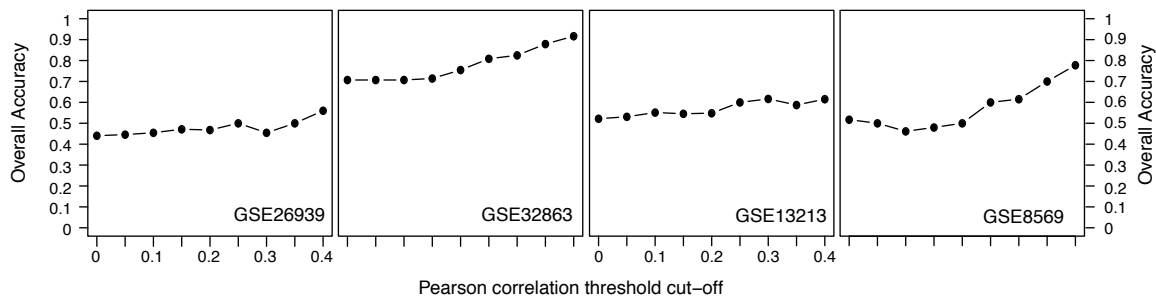**B)**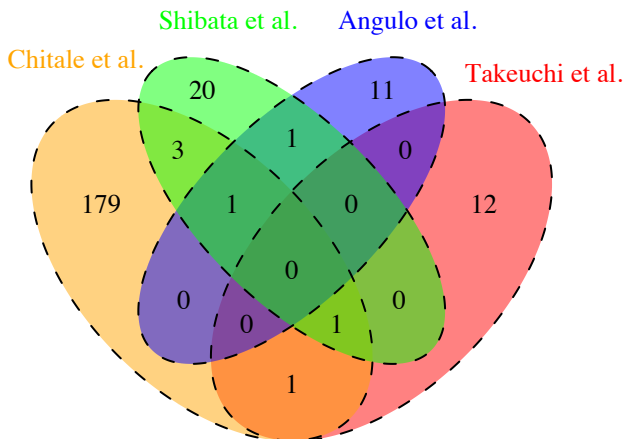**C)**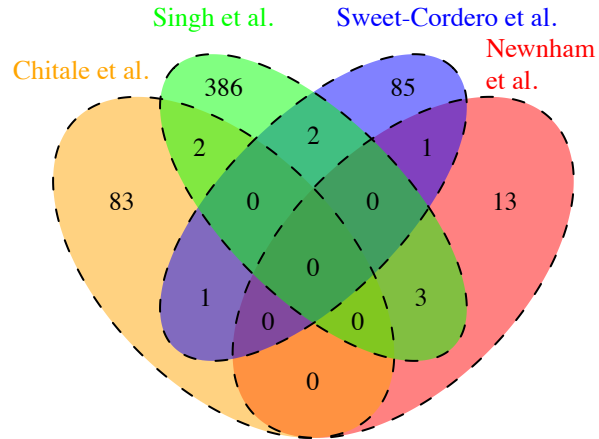

Supplement: Figure S3 — Comparison of public EGFR/KRAS signatures and classification by a set of genes differentially expressed between EGFR/KRAS mutation groups across multiple cohorts. (A) Overall accuracy for classification of four independent adenocarcinoma cohorts using a 96-gene centroid classifier. The number of genes in the centroid matching to the different cohorts varies. The x-axis shows Pearson correlation cut-off for assigning a sample to the centroid with the highest correlation. Increasing correlation cut-offs introduces growing numbers of unclassified samples, which are excluded in calculation of accuracy. (B) Venn-diagram of the gene overlap between four reported gene lists of differentially expressed genes between EGFR-mutated and EGFR-wild type adenocarcinoma tumors. (C) Venn-diagram of the gene overlap between two reported gene lists of differentially expressed genes between KRAS-mutated and KRAS-wild type adenocarcinoma tumors, and two reported KRAS mutant signatures [33,40]. (PDF) [file pone.0078614.s003.pdf]

**A)**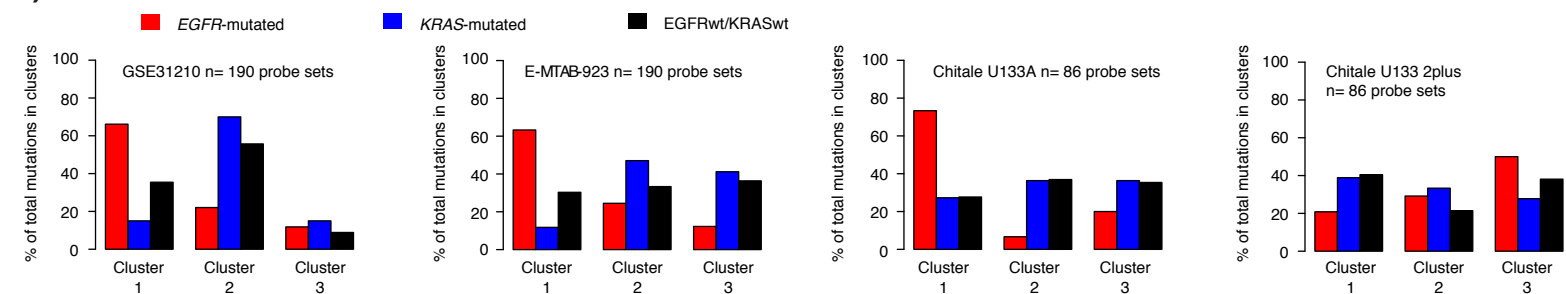**B)**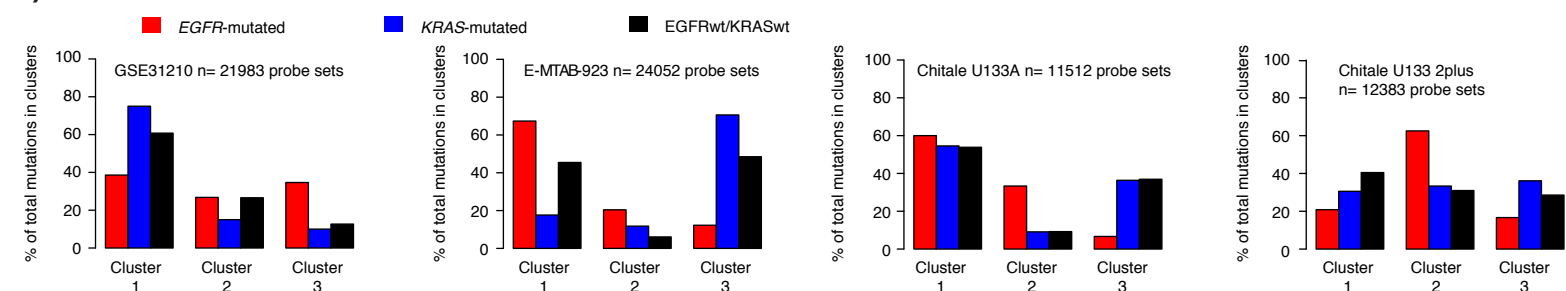**C)**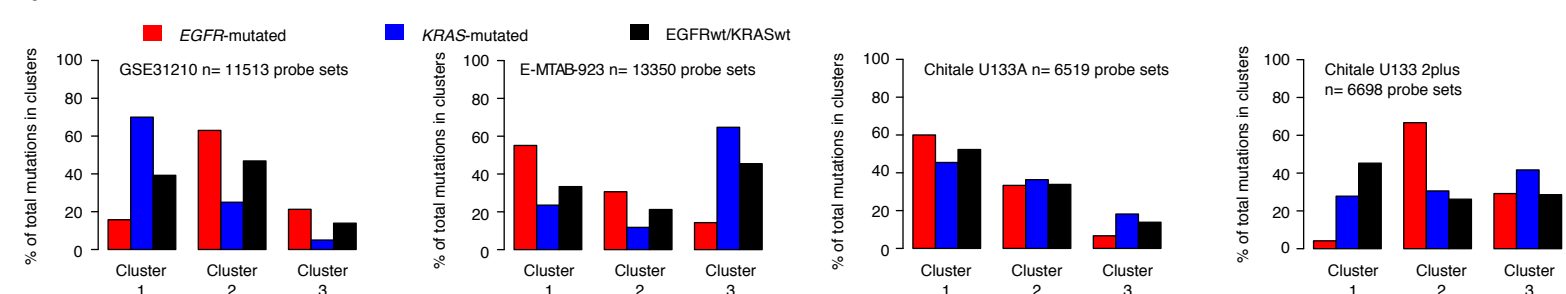**D)**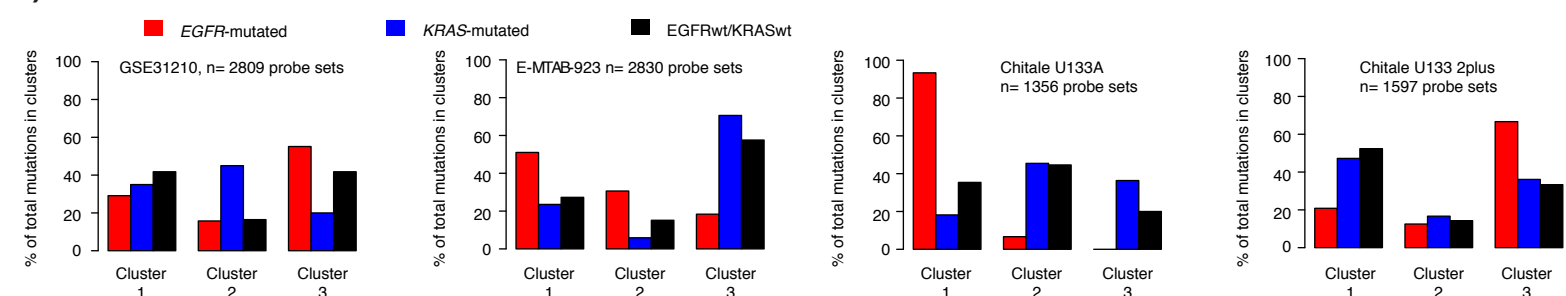**E)**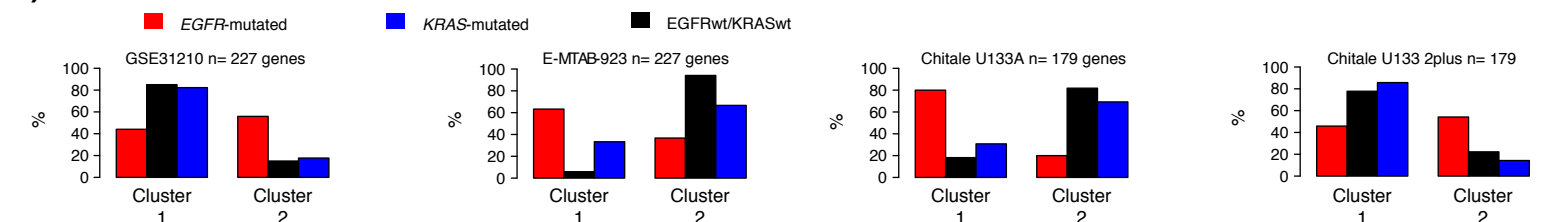

Supplement: Figure S4 — Unsupervised analyses of four Affymetrix adenocarcinoma gene expression cohorts using different probe sets. Unsupervised hierarchical clustering was performed using Pearson correlation and complete linkage using five different probe sets in four adenocarcinoma cohorts that were analyzed by Affymetrix gene expression microarrays. Dendrograms for each cluster tree were cut into the top two or three clusters, and the number of probe sets used in the clustering is shown for each cohort. For each cohort the distribution of EGFR-mutated (red), KRAS-mutated (blue), and EGFRwt/KRASwt (black) tumors are shown across clusters as bars. Percentages in bar plots correspond to, e.g., how many EGFR-mutated tumors of the total number of EGFR-mutated cases that reside in a particular cluster. (A) Clustering based on probe sets from a list of 190 probe sets reported to be upregulated in EGFRwt/KRASwt adenocarcinomas [26]. (B) Clustering based on probe sets with log2ratio standard deviation >0.3 across tumors in a cohort. (C) Clustering based on probe sets with log2ratio standard deviation >0.5 across tumors in a cohort. (D) Clustering based on probe sets with log2ratio standard deviation >1 across tumors in a cohort. (E) Clustering based on matching genes from the list of top 250 genes reported by Singh et al. [33]. Each dendrogram is cut into the top two clusters. Division of dendrograms into three groups did not identify KRAS-mutants as a single group without notable inclusion of EGFRwt/KRASwt tumors in all cohorts. (PDF) [file pone.0078614.s004.pdf]
