# Supplementary material for: Genomic and Transcriptional Alterations in Lung Adenocarcinoma in Relation to EGFR and KRAS Mutation Status
Source: PLoS One. 2013 Oct 24;8(10):e78614. doi: 10.1371/journal.pone.0078614 (PMC3812039; doi:10.1371/journal.pone.0078614)
Supplement: File S1 — Document with details concerning used analysis methods. (DOC) [file pone.0078614.s005.doc]

**Supplementary Methods**

**General information**

All statistical analysis and data processing steps described below were performed using R ([www.**r**-project.org/](http://www.r-project.org/)). All genomic data sets were matched to the hg18/NCBI36 genome build. Matching of genomic regions to genes was performed using the hg18 gene annotation object from GISTIC 2.0. Mutation status for samples in included genomic or mRNA data sets were identified from original publications, or by cross-referencing to other publications using the same samples, e.g. Ding et al. [1] for Weir et al. [2]. Explicit mutation information was not available for a majority of samples and consequently not included in analyses, or analyzed specifically.

This document outlines:

1. Generation of copy number and B allele estimates for genomic data sets.
2. Copy number segmentation and post-processing of segmented data for genomic data sets.
3. B allele frequency segmentation of SNP data sets.
4. Integration of copy number and B allele frequency segmented data, and merging of genomic data sets.
5. Quality assessment of samples from genomic data sets for inclusion in the study.
6. Merging of genomic data sets.
7. mGISTIC analysis of genomic data sets
8. Definition of recurrent amplifications in genomic data sets.
9. GAP-analysis of SNP data sets.
10. Statistical testing between subgroups of tumors – genomic regions.
11. Gene expression analyses.
12. *EGFR* / *KRAS* mutation analysis in GSE37745 and GSE28572.

**Estimation of copy number and allele estimates for Affymetrix SNP data**

Estimates of copy number (CN) and B Allele Frequency (BAF) were made in an unmatched fashion, without usage of any matched normal tissue (as such tissues were only available for parts of the Weir et al. and TCGA cohorts). For the different Affymetrix data sets CN and BAF estimates were generated using a combination of CRMAv2 [3] and ACNE [4] as outlined in the ACNE vignette (<http://www.aroma-project.org/>), and as further described in Staaf et al. [5].

Specifically, for the GSE36363 [6] data set samples were analyzed using either Affymetrix 6.0 or 250K Sty chips. For 250K Sty samples 276 HapMap cases obtained from GEO as series GSE5173 were used as specific reference set in the NmfSnpPlm function in ACNE to generate CN and BAF estimates. For Affymetrix 6.0 data 270 HapMap cases obtained from Affymetrix (www.affymetrix.com) were used as specific reference set in the NmfSnpPlm function in ACNE to generate CN and BAF estimates. After estimation of CN and BAF values, only SNP probes were used in further analyses, corresponding to approximately 931000 probes. The reason for exclusion of copy number probes present on the 6.0 chip is that no BAF estimates are calculated for these probes by ACNE.

**Processing of E-TABM-1169 (Illumina SNP beadchips)**

Normalized copy number and B allele frequency for cases analyzed by Illumina SNP beadchips were obtained from E-TABM-1169 [7,8]. Special consideration was required for analysis of E-TABM-1169 because this data set was analyzed on Illumina 370K SNP beadarrays and samples displayed a high degree of non-malignant infiltration. In combination with the generally lower log2ratio response to copy number alterations for the Illumina platform (see e.g. [9]) this caused problems with the fixed log2ratio thresholds used for calling copy number alterations. All samples in E-TABM-1169 were also analyzed by Agilent 244K arrays as a part of the E-TABM-926 data set from the same group/authors. Investigation of e.g. CN-FGA estimates showed a bias for considerably lower estimates, i.e., less detected copy number alterations, in Illumina samples compared to corresponding Agilent analyses, even after correction for tumor ploidy and normal cell contamination by GAP analysis (see below). To address this issue we merged the Illumina copy number data and corresponding Agilent data for samples in E-TABM-1169. Specifically, we started with GLAD segmented Illumina SNP profiles for cases in E-TABM-1169, post-processed and merged to the 3000bp probe set (see below). For each genomic segment in the Illumina data we updated the segmented log2ratio value for the segment to correspond to the dominant Agilent GLAD segmented value in the corresponding genomic region in the Agilent data for the same sample. In this process, all breakpoints between segments are still defined by the Illumina 370K analyses for samples in E-TABM-1169, only the individual segment copy numbers are updated. Prior to any update of segmented log2ratios we made sure, by visual inspection, that all pairs of genomic profiles (Illumina and Agilent for a specific sample) were centered similarly. After merging we visually inspected all cases. This procedure made E-TABM-1169 comparable to other data sets in e.g. fraction of the genome altered by copy number alteration.

**GLAD copy number segmentation and post processing of segmented data**

Prior to copy number segmentation genomic profiles were centralized similarly as described [10]. GLAD segmentation was performed using the Bioconductor GLAD [11] package for R ([www.bioconductor.org](http://www.bioconductor.org/)) as described [5]. Segmented profiles were post-processed as described [5]. Specifically for GSE36363 250K data segments < 5 probes or < 4000bp were joined with adjacent segments. For GSE36363 6.0 data corresponding cut-offs were < 15 probes or < 4000 bp in size.

**BAFsegmentation**

BAFsegmentation [12] was performed using the modified version reported by Staaf et al. [13] (available through the BAFsegmentation web-site) as described [5].

**Integration of partitioned CN and mBAF**

Post processed CN profiles and partitioned mBAF profiles (reflected BAF estimates, see [12]) for cases analyzed by SNP arrays were integrated using R scripts, with the aim to assign a partitioned mBAF value to an existing CN segment, and in certain instances to create new CN segments based on mBAF data as described [5].

**Quality assessment of genomic profiles**

Genomic profiles (copy number profiles plus B allele frequency plots when possible) were visually inspected for all cases, and cases with poor quality, inconsistent BAF and/or CN estimates were excluded. In addition, cases with flat copy number profiles were also excluded. Exclusion of cases with flat copy number profiles was based on that we did not find larger regions with apparently somatic copy neutral allelic imbalance in cases with a flat genomic profile analyzed by SNP arrays. Consequently, it appears difficult to conclude whether these cases in fact harbor tumor cells or not.

Moreover, the GSE34140 and GSE28572 data sets reports inclusion of micro- / macrodissected tissues to some extent (specific sample information is not available), while information for other data sets is missing.

**Merging of partitioned genomic profiles from different data sets**

Merging of partitioned genomic tumor profiles from different data sets into a 3000bp virtual probe set was performed as described [5].

**Post merging recentralization**

After merging all data sets to the common 3000bp probe set a final recentralization of copy number profiles were performed similarly as described [5,10].

**mGISTIC analysis**

Segmented CN profiles from adenocarcinoma cases merged to the common 3000bp probe set were subjected to a modified GISTIC algorithm (mGISTIC) for detection and delineation of significant copy number alterations as described [5]. Virtual probes overlapping reported CNVs were identified by matching probe positions to the ”variation.hg18.v9.mar.2010.txt” file downloaded from the Database of Genomic Variants (<http://projects.tcag.ca/variation/>) and subsequently removed prior to analysis. mGISTIC thresholds for gain and loss were set to log2ratio ±0.12. Parameters were identical to Staaf et al. [5] except for a p-value threshold of 0.1 and a higher delta.g.fraction (g-score boundary) value of 0.97 (0.96 in Staaf et al.). The more relaxed p-value threshold was chosen to allow possible identification of regions altered in a smaller subset of cases.

**mGISTIC permutation analysis**

Permutation analysis was performed to investigate the robustness of identified mGISTIC regions similarly as in Staaf et al. [5].

**Identification of recurrent amplifications in mGISTIC regions.**

For mGISTIC regions or individual genes the mean partitioned CN were computed for probes matching region/gene boundaries. A CN threshold of log2ratio >0.8 was used to identify samples with amplification for a specific region or gene similar to Weir et al. [2]. For a region to be called as recurrently amplified >7 cases were required to show amplification.

**GAP analysis**

GAP [14] analysis was performed using integrated CN and AI data merged to the common probe set as described above. R-scripts were used to transform the partitioned and integrated data into a format suitable for the “CopyNumber_and_Genotype” GAP function obtained from authors website [13]. The in silico tumor ploidy was calculated from the output of the above function as described [14]. Total LOH, copy-neutral LOH, and copy-neutral allelic imbalance were calculated from allele-specific copy numbers as described [5,15]. LOH implies an allele-specific copy number of 0 for one allele. Total LOH includes LOH caused by copy number loss and copy-neutral LOH. Copy-neutral allelic imbalance includes copy-neutral LOH by its definition but also genomic regions with uneven allele specific copy numbers (e.g. BBA) and copy neutral status. A typical example of a tumor with high fraction of copy-neutral allelic imbalance is a tumor with main ploidy = 3N.

**Statistical testing between subgroups of tumors – genomic regions**

Fisher's exact test or the Chi-square test was used to identify significantly different mGISTIC regions, recurrent amplifications, and previously reported regions between subgroups of tumors. For regions of gain and loss separately, samples were classified as having copy gain or not and copy loss or not, respectively, based on log 2 ratio thresholds of ±0.12. Two-by-two or two-by-three contingency tables tested gained versus no gain and loss versus no loss between subgroups. Similar approach was taken to test for differences in frequency of LOH, CNN-LOH and CNN-AI between subgroups of tumors.

**Gene expression analyses**

A total of 9 public gene expression data sets were analyzed (Chitale et al. was divided into two data sets due to different Affymetrix platforms). All Affymetrix data sets were normalized using GCRMA [16]. Normalized expression values for non-Affymetrix data sets were obtained from Gene Expression Omnibus [17]. Only tumor samples were selected, and gene expression differences are interpreted as differences in relative expression between tumors. All data sets were mean-centered for each probe across all samples. Prior to analyses we removed probes without a gene symbol. For Affymetrix data sets original probe set identifiers were used whenever possible. For duplicated probes, the probe with the highest standard deviation was kept. All matching of genes between different microarray platforms were performed using gene symbols or gene ids.

*Identification of a set of genes differentially expressed between mutation groups*

Prior to statistical analyses a variance filter was employed removing probes with standard deviation ≤0.3. For identification of a set of genes consistently differentially expressed between the three mutation groups (*EGFR*-mutated, *KRAS*-mutated, EGFRwt/KRASwt) we used ANOVA with false discovery rate (FDR) adjustment from the multtest R package [18] applied to five Affymetrix adenocarcinoma data sets (Chitale U133A, Chitale U133 2plus, E-MTAB-923, GSE37745, and GSE31210). Probes with FDR < 0.05 were considered statistically significant. A final list was generated including genes differentially regulated in 4 out of 5 analyzed data sets. Gene expression centroids were created based on the genes consistently differentially expressed in 4 out of 5 data sets. The centroid classifier was chosen due to the large number of different array platforms analyzed. Centroid values for a gene were calculated by first calculating the median value of the gene in each class (*EGFR*-mutated, *KRAS*-mutated, EGFRwt/KRASwt) for each data set individually (for each gene rendering 5 median values for each mutation group in the five data sets). Next we calculated the mean of median values for all data sets were the gene was differentially expressed (e.g. mean of 4 medians for each mutation group if a gene was differentially expressed in 4 data sets). Thus centroid values represent the “mean of medians” for each gene. Centroids were applied to independent data sets (GSE26939, GSE32863, GSE8569, GSE13213) analyzed by different microarray platforms for classification of tumors using Pearson correlation. Samples were assigned to a centroid based on a Pearson correlation cut-off that was varied between 0 and 0.4. Higher correlation cut-offs introduced larger number of unclassified samples that were excluded in calculations of sensitivity and specificity. The number of genes in the centroid matching to the different data sets varied due to differences in the content of the different microarray platforms.

*Unsupervised class analysis*

Unsupervised class analysis was performed using hierarchical clustering with Pearson correlation and complete linkage for Chitale U133A, Chitale U133 2plus, GSE31210, and E-MTAB-923. Analysis was performed for each data set separately on a) probes matching the Okayama et al. [19] 190-probe set, b) a KRAS dependency signature [20], and c) normalized and variance filtered data (using thresholds log2 standard deviation 0.3, 0.5 and 1) for samples with available mutation status. Hierarchical trees were divided into the top two or three clusters.

***EGFR* / *KRAS* mutation analysis in GSE37745**

*DNA extraction*

A haematoxylin-eosin stained 4-μm tape-transfer section (CryoJane, Instrumedics, Richmond, IL, U.S.) was prepared from each fresh frozen OCT-embedded tumor sample and thoroughly examined by a pathologist to ensure that the sample contained representative tumor tissue and more than 50% tumor cells. Depending on the size of the tissue sample, DNA was extracted from 2-10 frozen tissue sections (10μm) using the QIAamp DNA Mini Kit (Qiagen, Hilden, Germany) according to the manufacturer´s protocol and DNA concentrations were measured using a ND-1000 spectrophotometer (NanoDrop Technologies, Wilmington, DE, U.S.).

*Sanger sequencing (EGFR)*

PCR amplification of EGFR exon 18-21 was performed using the GeneAmp 2700 PCR cycler (Applied Biosystems) and the ready-to-use ABgene PCR Master Mix (Thermo Fisher Scientific, Waltham, MA). Fifteen ng of DNA and 200nM of forward and reverse primer were added to the master mix in a total volume of 25μL. PCR conditions for exon 18, 19 and 21 were as follows: 40°C for 10 min, 95°C for 10 min, 35 cycles of 94°C for 30 sec, 59°C for 20 sec, and 72°C for 45 sec, followed by 72°C for 7 min. Conditions for the exon 20 PCR were 95°C for 10 min, followed by 35 cycles of 95°C for 20 sec, 66°C for 20 sec, and 72°C for 45 sec, followed by a final step of 72°C for 5 min. PCR products were separated on a 2% agarose gel and visualized by ethidium bromide staining, purified using exonuclease I and shrimp alkaline phosphatase, and sequenced in both forward and reverse direction using BigDye Terminator v1.1 (Applied Biosystems) on the 3130xl Genetic Analyzer (Applied Biosystems). The same primers as in the initial PCR reactions were used in the sequencing reaction. Results indicating a mutation were confirmed on a new PCR product in a second independent analysis.

Primer sequences EGFR Sanger sequencing exon 18-21:

| Amplicon | Forward primer (5´–3´) | Reverse primer (5´–3´) | Length |
| --- | --- | --- | --- |
| EGFR:exon18 | GACCCTTGTCTCTGTGTTCTTGT | TATACAGCTTGCAAGGACTCTGG | 241 bp |
| EGFR:exon19 | CACAATTGCCAGTTAACGTCTTC | AGGGTCTAGAGCAGAGCAGC | 231 bp |
| EGFR:exon20 | GATCGCATTCATGCGTCTTCACC | TTGCTATCCCAGGAGCGCAGACC | 362 bp |
| EGFR:exon21 | CATGATGATCTGTCCCTCACAG | CTGGTCCCTGGTGTCAGGAA | 249 bp |

*Pyrosequencing (KRAS)*

Pyrosequencing and the PyroMark Q24 KRAS Kit (Qiagen) was used to detect mutations in KRAS codon 12/13 (exon 2) and 61 (exon 3). Separate PCR reactions for codon 12/13 and 61 were performed on the GeneAmp 2700 PCR cycler (Applied Biosystems) in a total volume of 50μl containing 10ng of DNA, 1.5mM MgCl_2_, 200μM of each dNTP, 0.025u/μL Platinum Taq DNA Polymerase (Invitrogen), and 200nM of the forward and reverse primer included in the PyroMark Q24 KRAS Kit (Qiagen). PCR conditions were as follows: 95°C for 15 min, 45 cycles of 95°C for 20 sec, 58°C for 20 sec, and 72°C for 20 sec, followed by a final elongation step of 72°C for 5 min. Twenty μL of the PCR product was sequenced on the Pyrosequencing Q24 System (Qiagen) according to the manufacturer’s instructions using Streptavidin Sepharose High Performance (GE Healthcare, Uppsala, Sweden), PyroMark Gold Q96 reagents (Qiagen), and sequencing primers for codon 12/13 and 61 respectively provided in the PyroMark Q24 KRAS Kit (Qiagen). Results indicating a mutation were confirmed on a new PCR product in a second independent analysis.

**References**
