# Supplementary material for: Genomic and Transcriptional Alterations in Lung Adenocarcinoma in Relation to EGFR and KRAS Mutation Status
Source: PLoS One. 2013 Oct 24;8(10):e78614. doi: 10.1371/journal.pone.0078614 (PMC3812039; doi:10.1371/journal.pone.0078614)
Supplement: Table S4 — Analysis of genomic regions reported in the literature to stratify mutation groups in the current cohort. (DOC) [file pone.0078614.s009.doc]

**Table S4. Analysis of reported genomic regions stratifying adenocarcinomas based on *EGFR* and/or *KRAS*** mutation status.

| **Study** | **Region** | **Chr** | **Start^A^**  **(bp)** | **Stop^A^**  **(bp)** | **Size**  **(Mb)** | **Overlapping mGISTIC region^B^** | **Reference comparison^C^** | **Bonferroni**  **P-value reference comparison^D^** | **Bonferroni**  **P-value**  **EGFR-mut/**  **KRAS-mut/**  **EGFRwt/KRASwt ^D^** |
| --- | --- | --- | --- | --- | --- | --- | --- | --- | --- |
| Fong (1) | Gain_1q23-q31 | 1 | 154800001 | 197500000 | 42.7 | Amp_1q24.2 | EGFR+ vs wt | NS | NS |
| Fong | Gain_6p12-p21.1 | 6 | 40600001 | 57200000 | 16.6 | Amp_6p21.1 | EGFR+ vs wt | NS | NS |
| Fong | Gain_7q11.2 | 7 | 61100001 | 77400000 | 16.3 | **Amp_7q11.21** | EGFR+ vs wt | **0.0004** | **0.001** |
| Fong | Loss_3p21 | 3 | 43600001 | 54400000 | 10.8 |  | EGFR+ vs wt | NS | NS |
| Fong | Loss_8p23-p22 | 8 | 1 | 19100000 | 19.1 | **Del_8p23.2-p23.1** | EGFR+ vs wt | **8e-5** | **1e-4** |
| Fong | Loss_9q33 | 9 | 116700001 | 129300000 | 12.6 |  | EGFR+ vs wt | 0.003 | 0.02 |
| Fong | Loss_10q25 | 10 | 105700001 | 119100000 | 13.4 |  | EGFR+ vs wt | 0.0004 | **0.002** |
| Fong | Loss_13q13 | 13 | 31100001 | 39500000 | 8.4 |  | EGFR+ vs wt | 0.02 | NS |
| Blons (2) | Gain_14q21.3 | 14 | 46489396 | 47088012 | 0.6 |  | EGFR+ vs KRAS+ | 0.01 | 0.03 |
| Blons | Gain_7p21.3-p21.2 | 7 | 11727815 | 13456320 | 1.73 |  | EGFR+ vs KRAS+ | **0.0002** | **1e-7** |
| Blons | Gain_7p21.3 | 7 | 11120441 | 11377580 | 0.26 |  | EGFR+ vs KRAS+ | **0.0003** | **3e-7** |
| Blons | Gain_7p21.2-p15.3 | 7 | 18006698 | 20386036 | 2.38 |  | EGFR+ vs KRAS+ | **4e-5** | **1e-9** |
| Newnham (3) | Loss_1p36.32-p13.2 | 1 | 2300001 | 115900000 | 113.6 | Del_1p36.12, Del_1p13.3 | KRAS+ vs wt | NS | NS |
| Newnham | Loss_6q11.1-q27 | 6 | 60500001 | 170899992 | 110.4 | Del_6q13, **Del_6q16.3-q21**, Del_6q27 | KRAS+ vs wt | 0.006 | 0.02 |
| Newnham | Loss_11p13-q13.2 | 11 | 31000001 | 69200000 | 38.2 |  | KRAS+ vs wt | NS | NS |
| Newnham | Loss_11q21-qter | 11 | 92300001 | 134452384 | 42.2 | Del_11q24.3 | KRAS+ vs wt | NS | NS |
| Newnham | Loss_12pter-p13.1 | 12 | 1 | 14800000 | 14.8 | Del_12p13.1 | KRAS+ vs wt | NS | NS |
| Newnham | Gain_1q21.1-q43 | 1 | 142400001 | 241700000 | 99.3 | Amp_1q21.1, Amp_1q21.2, Amp_1q21.3-q22, Amp_1q24.2, Amp_1q32.1 | KRAS+ vs wt | NS | NS |
| Broet (4) | Gain_1p36 | 1 | 1 | 27800000 | 27.8 |  | EGFR+ vs wt | **7e-9** | **4e-8** |
| Broet | Gain_1p35 | 1 | 27800001 | 34400000 | 6.6 |  | EGFR+ vs wt | **1e-7** | **5e-9** |
| Broet | Gain_7p22-p21 | 7 | 1 | 19500000 | 19.5 | **Amp_7p22.3-p22.2, Amp_7p21.1** | EGFR+ vs wt | **9e-9** | **1e-8** |
| Broet | Gain_7p15-p12 | 7 | 19500001 | 53900000 | 34.4 |  | EGFR+ vs wt | **5e-10** | **3e-9** |
| Broet | Gain_16p13 | 16 | 1 | 16700000 | 16.7 | **Amp_16p13.13** | EGFR+ vs wt | **5e-9** | **1e-8** |
| Broet | Gain_16p12 | 16 | 16700001 | 27600000 | 10.9 |  | EGFR+ vs wt | **4e-7** | **8e-7** |
| Broet | Gain_14q31-q32 | 14 | 78400001 | 106368585 | 28 |  | EGFR+ vs wt | 0.001 | 0.002 |
| Broet | Loss_21q21-q22 | 21 | 15300001 | 46944323 | 31.6 | Del_21q21.1 | wt vs EGFR+ | 0.006 | **0.001** |
| Reinmuth (5) | Gain_7p22 | 7 | 1 | 7200000 | 7.2 | **Amp_7p22.3-p22.2** | EGFR+ vs wt | **1e-6** | **3e-6** |
| Reinmuth | Gain_7p14-p11 | 7 | 31800001 | 59100000 | 27.3 | **Amp_7p11.2** | EGFR+ vs wt | **5e-11** | **3e-10** |
| Reinmuth | Gain_7q33-q36 | 7 | 132400001 | 158821424 | 26.4 |  | EGFR+ vs wt | **0.0006** | **0.002** |
| Reinmuth | Loss_8p12 | 8 | 29700001 | 38500000 | 8.8 | **Del_8p21.2-p12** | EGFR+ vs wt | **3e-7** | **6e-7** |
| Reinmuth | Loss_8p23-p21 | 8 | 1 | 29700000 | 29.7 | **Del_8p23.2-p23.1, 8p21.2-p12** | EGFR+ vs wt | **6e-6** | **1e-5** |
| Reinmuth | Loss_6q24 | 6 | 139100001 | 149100000 | 10 |  | wt vs EGFR+ | NS | NS |
| Reinmuth | Loss_17p13 | 17 | 1 | 11200000 | 11.2 | Del_17p13.1 | wt vs EGFR+ | NS | NS |
| Reinmuth | Gain_21q11-q22 | 21 | 12300001 | 46944323 | 34.6 |  | EGFR+ vs wt | 0.0002 | 0.0007 |

A: Coordinates in hg18 build.

B: mGISTIC regions in bold found to differentiate between *EGFR*-mutated (EGFR+), *KRAS*-mutated (KRAS+), and EGFRwt/KRASwt tumors in the current study.

C: Type of comparison in original study. The frequency of a region is highest in the first group vs the second, e.g., EGFR+ vs wt means higher frequency of an alteration in EGFR-mutated tumors compared to EGFR-wild type tumors. *EGFR*-mutated (EGFR+), *KRAS*-mutated (KRAS+). No study subgroups tumors into all three groups, consequently wt corresponds to *EGFR*-wild type tumors when present with EGFR+ and *KRAS*-wild type tumors when present with KRAS+.

D: Bonferroni adjusted Fisher’s exact p-value. Regions with p-values in bold also display >20% maximum frequency difference between tested groups in the correct orientation as reported in original study. For tested regions a call of gain or loss was given if at least 50% of probes showed alteration, using cut-offs of ±0.12 in log2ratio as thresholds. NS = Not significant (p>0.05).

References:

1. Fong Y, Lin YS, Liou CP, Li CF, Tzeng CC. Chromosomal imbalances in lung adenocarcinomas with or without mutations in the epidermal growth factor receptor gene. Respirology 2010;15: 700-5.

2. Blons H, Pallier K, Le Corre D, Danel C, Tremblay-Gravel M, Houdayer C*, et al.* Genome wide SNP comparative analysis between EGFR and KRAS mutated NSCLC and characterization of two models of oncogenic cooperation in non-small cell lung carcinoma. BMC Med Genomics 2008;1: 25.

3. Newnham GM, Conron M, McLachlan S, Dobrovic A, Do H, Li J*, et al.* Integrated mutation, copy number and expression profiling in resectable non-small cell lung cancer. BMC cancer 2011;11: 93.

4. Broet P, Dalmasso C, Tan EH, Alifano M, Zhang S, Wu J*, et al.* Genomic profiles specific to patient ethnicity in lung adenocarcinoma. Clin Cancer Res 2011;17: 3542-50.

5. Reinmuth N, Jauch A, Xu EC, Muley T, Granzow M, Hoffmann H*, et al.* Correlation of EGFR mutations with chromosomal alterations and expression of EGFR, ErbB3 and VEGF in tumor samples of lung adenocarcinoma patients. Lung Cancer 2008;62: 193-201.
